# Tiara-like Hexanuclear Nickel–Platinum Alloy Nanocluster

**DOI:** 10.1021/acs.jpclett.3c03594

**Published:** 2024-02-01

**Authors:** Tomoshige Okada, Tokuhisa Kawawaki, Kana Takemae, Shiho Tomihari, Taiga Kosaka, Yoshiki Niihori, Yuichi Negishi

**Affiliations:** † Department of Applied Chemistry, Faculty of Science, Tokyo University of Science, 1−3 Kagurazaka, Shinjuku-ku, Tokyo 162−8601, Japan; ‡ Research Institute for Science and Technology, Tokyo University of Science, 2641 Yamazaki, Noda, Chiba 278−8510, Japan

## Abstract

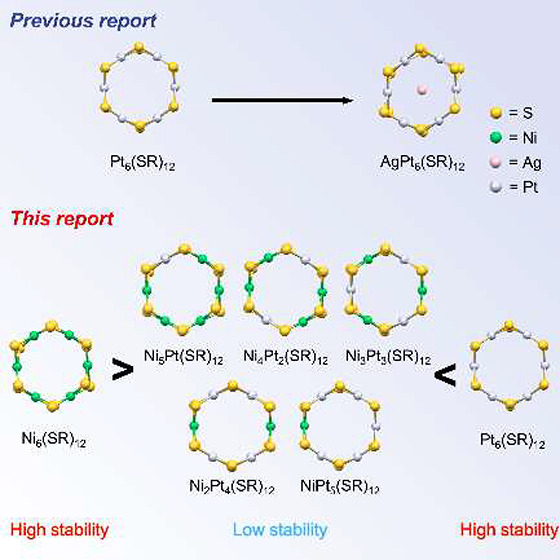

Tiara-like metal nanoclusters (TNCs) have attracted a great deal of attention because of their high stability and easy synthesis under atmospheric conditions as well as their high activity in various catalytic reactions. Alloying is one of the methods that can be used to control the physicochemical properties of nanoclusters, but few studies have reported on alloy TNCs. In this study, we synthesized alloy TNCs [Ni_*x*_Pt_6–*x*_(PET)_12_, where *x* = 1–5 and PET = 2-phenylethanethiolate] consisting of thiolate, nickel (Ni), and platinum (Pt). We further evaluated the stability, geometric structure, and electronic structure by high-performance liquid chromatography and density functional theory calculations. The results revealed that Ni_*x*_Pt_6–*x*_(PET)_12_ has a distorted structure and is therefore less stable than single-metal TNCs.

Multinuclear metal complexes, termed metal nanoclusters (NCs), exhibit physicochemical properties different from those of bulk metals that are composed of the same metal elements.^1−17^ In particular, tiara-like metal NCs (TNCs) are ring compounds containing metal ions and can be synthesized in air for the group 10 metals, such as nickel (Ni),^18−24^ palladium (Pd),^25−31^ and platinum (Pt).^30−33^ They are widely used in a variety of fields, including catalysis, perhaps in part because of their relative ease of handling.^34−39^ Alloying enables precise control of the electronic and geometric structure of NCs, thus facilitating further functionalization. Therefore, alloying with various metals has been reported in many cases, especially for thiolate (SR)-protected gold (Au) NCs [Au_*n*_(SR)_*m*_] and silver (Ag) NCs [Ag_*n*_(SR)_*m*_].^3,40,41^ For TNCs, those encapsulating various metals and molecules can also be synthesized.^19,20,30,42−44^ For instance, in alloy TNCs with Ag(I) encapsulation by Pt_6_(SR)_12_, AgPt_6_(SR)_12_ (SR = 1-octanethiolate; SC_8_H_17_, 1-dodecanethiolate; SC_12_H_25_) has been reported with a new optical transition orbit based on charge transfer between Ag and Pt,^42^ and the luminescence quantum yield is substantially enhanced by the encapsulation (Figure 1A).^45^ However, the number of different elements that can be alloyed in TNCs is only one atom encapsulated in the ring structure. If TNCs can be alloyed with a larger number of different metals, it is expected that TNCs with more diverse chemical compositions can be obtained, which will enable more precise control of the electronic and geometric structures as well as an improvement in their optical properties and catalytic functions. TNCs can also be separated by methods such as thin-layer chromatography, gel permeation chromatography, and high-performance liquid chromatography (HPLC) to obtain TNCs with a single chemical composition.^25,26,32^ These high-resolution separation methods have been developed for atomic-precision separation of Au_*n*_(SR)_*m*_.^46^ Thus, it is now possible to separate Au_*n*_(SR)_*m*_ and their alloy NCs by not only size but also different ligands, alloy chemical compositions, isomers, and charge states.^46^

**Figure 1:**
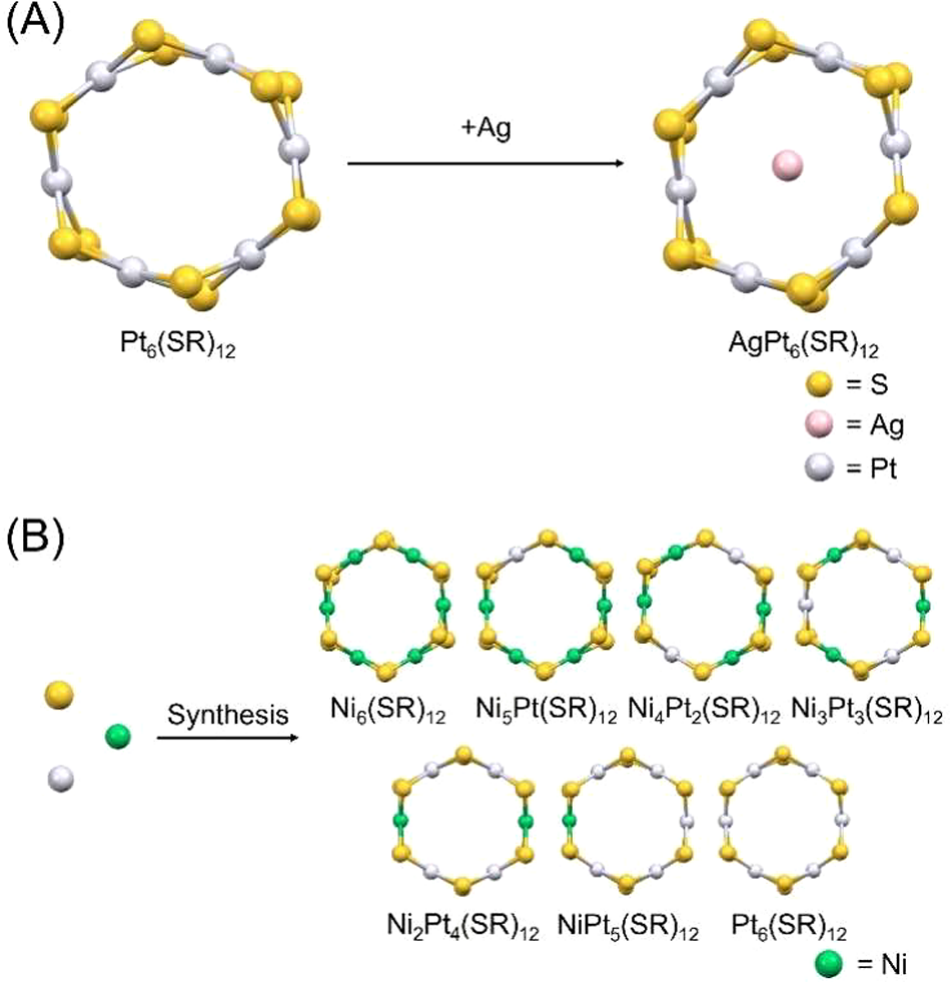
Schematic of (A) previous work^42,45^ and (B) this study for alloying of TNCs.

In this study, we reveal that Ni_*x*_Pt_6–*x*_(PET)_12_ (*x* = 1–5, and PET = 2-phenylethanethiolate), a NiPt alloy TNC, is a synthesizable alloy TNC and can be substituted by up to five atoms (Figure 1B). Density functional theory (DFT) calculations indicate that as the number of Pt substitutions increases, the absorption peak at ∼420 nm shifts, mainly because of a change in the highest occupied molecular orbitals (HOMOs) and lowest unoccupied molecular orbitals (LUMOs). We examined their stabilities on the basis of the geometrical structures predicted by DFT calculations. The results implied that alloys Ni_*x*_Pt_6–*x*_(SR)_12_ (*x* = 1–5) has a distorted structure, and hence, the NiPt alloy TNC presumably has a relatively low stability compared with those of Ni_6_(SR)_12_ and Pt_6_(SR)_12_. High-resolution separation by HPLC indicates that Ni_*n*_(PET)_2*n*_ (*n* = 4–6) can be separated, whereas Ni_*x*_Pt_6–*x*_(PET)_12_ (*x* = 1–5) is difficult to isolate because of ongoing metal exchange between TNCs in solution.

We synthesized product **1** [Ni_*x*_Pt_6–*x*_(PET)_12_ (*x* = 0–6)] by dissolving nickel nitrate hexahydrate [Ni(NO_3_)_2_·6H_2_O] and hydrogen hexachloroplatinate(IV) hexahydrate (H_2_PtCl_6_·6H_2_O) in 1-propanol and then adding 2-phenylethanethiol and triethylamine as a reducing agent. Figure 2 shows a matrix-assisted laser desorption ionization (MALDI) mass spectrum of **1**. In this case, we ionized **1** by adding Ag^+^ during MALDI mass spectrometry.^47^Figure 2 indicates that **1** exhibits peaks in positive-ion mode with mass-to-charge ratios (*m*/*z*) of 2242, 2379, 2516, 2653, and 2790. The isotope-derived peak intervals of these peaks are *m*/*z* 1, indicating that **1** was observed as monocations. The interval between these peaks is *m*/*z* ∼137, which is equal to the difference in molecular weight (Mw) between Ni (Mw = 58) and Pt (Mw = 195). The isotopic patterns of these peaks are in good agreement with those of [Ni_*x*_Pt_6–*x*_(PET)_12_ + Ag]^+^ (*x* = 1–5) (Figure 2). In addition, when performing a similar experiment by changing the SR ligands to 1-propanethiolate [**2**; Ni_*x*_Pt_6–*x*_(SC_3_H_7_)_12_], 1-octanethiolate [**3**; Ni_*x*_Pt_6–*x*_(SC_8_H_17_)_12_], and 1-dodecanethiolate [**4**; Ni_*x*_Pt_6–*x*_(SC_12_H_25_)_12_], we obtained MALDI mass spectra with an interval of *m*/*z* ∼137 attributable to [Ni_*x*_Pt_6–*x*_(SR)_12_ + Ag]^+^ (*x* = 0–6, and SR = SC_3_H_7_, SC_8_H_17_, or SC_12_H_25_) (Figure S1). In the transmission electron microscopy images of **1**, the monodispersed TNC with a small size (0.7 ± 0.1 nm) was observed (Figure S2). These results indicate that Ni_*x*_Pt_6–*x*_(SR)_12_ (*x* = 1–5), in which Ni is substituted with Pt, were obtained by this synthetic method.

**Figure 2:**
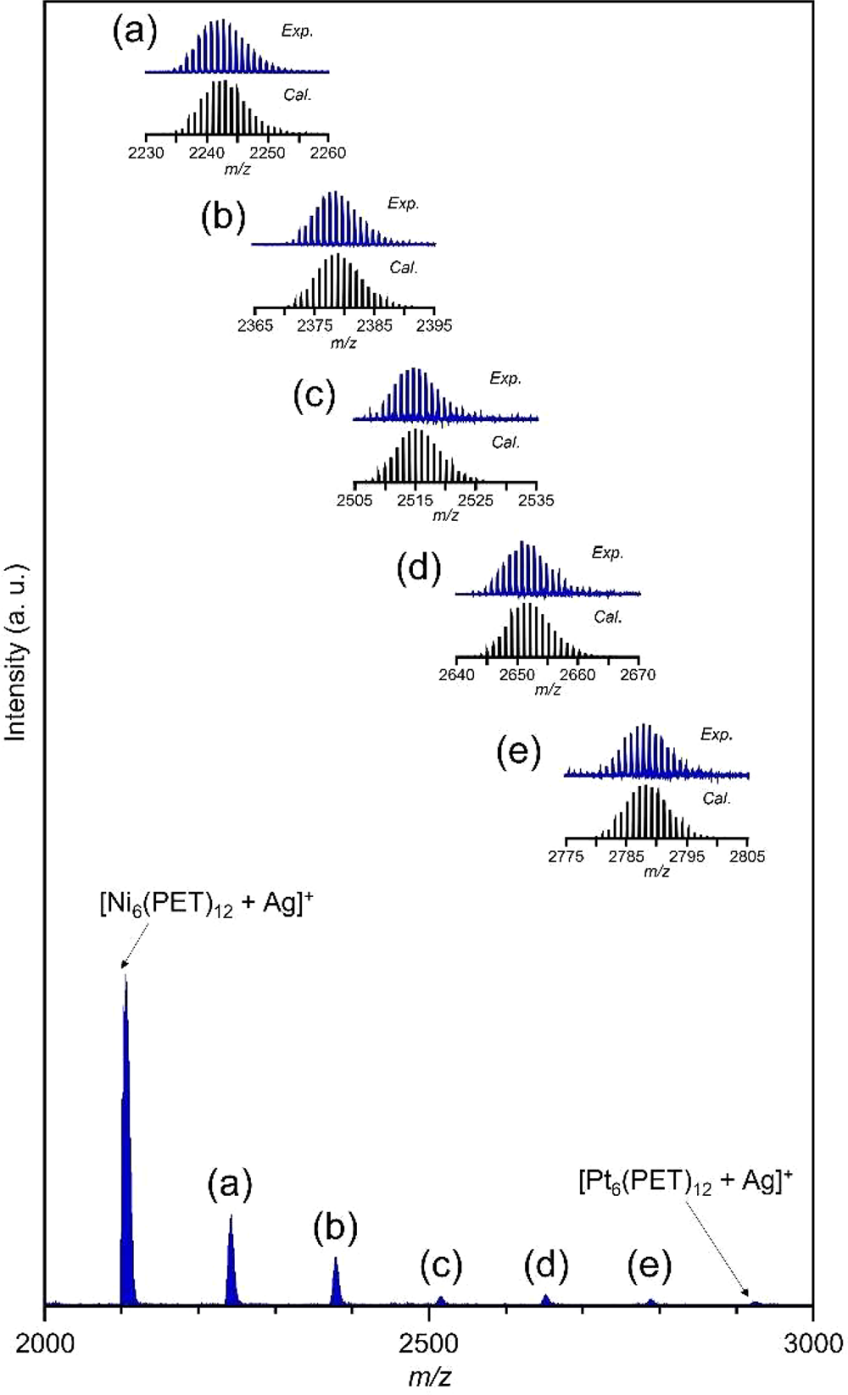
Positive-ion MALDI mass spectrum of **1**. Insets show a comparison of the isotope pattern between the experimental and calculated spectra of (a) [Ni_5_Pt(PET)_12_ + Ag]^+^, (b) [Ni_4_Pt_2_(PET)_12_ + Ag]^+^, (c) [Ni_3_Pt_3_(PET)_12_ + Ag]^+^, (d) [Ni_2_Pt_4_(PET)_12_ + Ag]^+^, and (e) [NiPt_5_(PET)_12_ + Ag]^+^.

We evaluated the electronic structure of **1** by X-ray absorption spectroscopy measurements at the Ni K- and Pt L_3_-edges and X-ray photoelectron spectroscopy (XPS). For Ni, we observed an electronic state close to Ni^2+^ from the pre-edge peak in the Ni K-edge X-ray absorption near-edge structure (XANES) spectrum and Ni 2p_3/2_ XPS spectra of **1** (Figures S3A and S4A).^36^ In the Ni K-edge Fourier-transform extended X-ray absorption fine structure (FT-EXAFS) spectra (Figure S5A), we observed no peak attributable to a Ni–Ni bond (∼2.1 Å) and only a peak attributable to a Ni–S bond (∼1.8 Å). In addition, the Pt L_3_-edge XANES and FT-EXAFS spectra and Pt 4f_7/2_ XPS spectra (Figure S4B) implied that Ni_*x*_Pt_6–*x*_(PET)_12_ (*x* = 1–5) has an electronic state close to Pt^2+^; the slight increase in absorption at the white line was observed in the XANES spectrum of **1** (Figure S3B), and only a peak (∼1.9 Å) attributable to the strong Pt–S bond was observed in the FT-EXAFS spectrum (Figure S5B).^48^ These results indicate that Ni and Pt atoms are directly bonded to S in PET in Ni_*x*_Pt_6–*x*_(PET)_12_ (*x* = 1–5).

The measurements of electrocatalytic activity (Figure S6) revealed that the hydrogen evolution activity of **1** is higher than that of pure Ni_6_(PET)_12_. The optical absorbance spectrum of **1** was slightly different from that of Ni_6_(PET)_12_ (Figure S7A), although both exhibited spectra overall similar to each other.

Because metal atoms are exposed to the outside in such Ni_*x*_Pt_6–*x*_(SR)_12_, the number of metal dopings is expected to affect the polarization of the TNCs as well as their electronic structures. Therefore, we tried to separated Ni_*x*_Pt_6–*x*_(SR)_12_ by reversed-phase (RP) HPLC by the differences in the polarity of each TNC.^46^Figures S7B and S8 show the obtained chromatograms of **1** and Ni_*n*_(PET)_2*n*_ (*n* = 4–6), which we roughly isolated in advance by thin-layer chromatography, by changing the mobile phase continuously from pure acetonitrile to a pure acetone using the gradient program. In the chromatogram of **1**, several strong peaks (**i**–**iii**) were observed (Figure S7B). Peak **i** agreed well with the retention time (*t*_R_) of Ni_4_(PET)_8_ (*t*_R_ = 14.6 min), and the MALDI mass spectrum of the sample fractionated at peak **i** confirmed that it was Ni_4_(PET)_8_ (Figure S8). Similarly, peaks **ii** (*t*_R_ = 16.2 min) and **iii** (*t*_R_ = 17.9 min) were attributable to Ni_5_(PET)_10_ and Ni_6_(PET)_12_, respectively (Figure S8). The strong peak of Ni_5_(PET)_10_ (peak **ii**) observed in the chromatogram of **1** is presumably due to the formation of Ni_5_(PET)_10_ by decomposition of the unstable species generated during the formation of Ni_*x*_Pt_6–*x*_(PET)_12_. In fact, we observed a trace quantity of Ni_5_(PET)_10_ (peak **ii**) in product **5** [Ni_*x*_Pt_6–*x*_(PET)_12_ (*x* = 0–6)] via a metal exchange reaction, in which we attempted to synthesize Ni_*x*_Pt_6–*x*_(PET)_12_ by metal exchange, by adding H_2_PtCl_6_ to previously isolated Ni_6_(PET)_12_ (Figure S9). This also implies that Ni_5_(PET)_10_ is a byproduct during the formation of Ni_*x*_Pt_6–*x*_(PET)_12_. From these results, one can consider that Ni_*x*_Pt_6–*x*_(SR)_12_ was produced by replacement of some Ni atoms in Ni_6_(SR)_12_ with Pt atoms while maintaining their geometrical structure.^49−51^ Also, there is the possibility that the Ni(SR)_*x*_ unit of Ni_6_(SR)_12_ was replaced by Pt(SR)_*x*_ via an interunit SN_2_-type reaction, leading to the formation of Ni_*x*_Pt_6–*x*_(SR)_12_.^52^

Next, we fractionated peak **v** (*t*_R_ = 20.3 min), which gave a relatively high intensity among peaks **iv**–**xi**, and evaluated the MALDI mass spectrum (Figure S10). We observed peaks attributable to Ni_*x*_Pt_6–*x*_(PET)_12_ at *m*/*z* 2242, 2379, 2516, 2653, and 2790, which we did not observe in peaks **i**–**iii**. This means that Ni_*x*_Pt_6–*x*_(PET)_12_ was included in peak **v**. However, peak **v** included various TNCs with different metal exchange numbers, and thereby, we could not observe a single chemical composition of Ni_*x*_Pt_6–*x*_(PET)_12_. Therefore, for peak **v**, we again introduced the fractionated sample into the RP-HPLC instrument to examine its purity. We observed multiple peaks attributable to various TNCs for fractionated peak **v** (Figure S11). This might be due to the progress of metal exchange^51^ between TNCs in the fractionated solution. Thus, Ni_*x*_Pt_6–*x*_(PET)_12_ is more unstable than Ni_*n*_(PET)_2*n*_ (*n* = 4–6), which is composed of a single metal species, and metal exchange and decomposition occur between TNCs in solution after fractionation. A similar trend has been reported for alloy NCs such as Au_*n*–*x*_Ag_*x*_(SR)_*m*_.^51^ Unfortunately, the other peaks, **iv** and **vi**–**xi**, could not be characterized in a fractionated manner because of their low synthesis yields (Figure S7B).

We also attempted RP-HPLC separations for products **2**–**4**, which were alloy TNCs synthesized with the different SR ligands (SC_3_H_7_, SC_8_H_17_, and SC_12_H_25_, respectively). In general, the longer the length of the alkyl chain, the poorer the separation performance; we also obtained the peaks at a retention time different from that of Ni_*n*_(SR)_2*n*_ (*n* = 4–6), suggesting that Ni_*x*_Pt_6–*x*_(SR)_12_ (SR = SC_3_H_7_, SC_8_H_17_, or SC_12_H_25_) could be separated from Ni_6_(SR)_12_ (Figure S12).

We studied the geometric and electronic structures of the obtained Ni_*x*_Pt_6–*x*_(PET)_12_ (*x* = 1–5) by DFT calculations to elucidate their stable structures and the origin of the optical absorption spectrum. In the calculations, we used methanethiolate (MT) instead of PET as the SR ligand for the sake of simplicity. Initially, we performed structural optimization of Ni_6_(MT)_12_ based on the geometric structure of Ni_6_(PET)_12_ obtained by single-crystal X-ray diffraction. As a result, we obtained a tiara-like structure in which methyl groups are arranged alternately horizontally as well as vertically from the upper and lower S of the Ni_6_-ring structure (Figure S13). Thus, changing the ligand from PET to MT did not substantially change the overall geometric structure of Ni_6_(SR)_12_.^35^

Next, we attempted to calculate Ni_*x*_Pt_6–*x*_(MT)_12_ (*x* = 1–6) by substituting Ni in Ni_6_(MT)_12_ with Pt; Figure S13 shows the optimized structure. For Ni_*x*_Pt_6–*x*_(MT)_12_ (*x* = 2–4), three isomers are expected to exist for each; thus, we performed structural optimization for them, as well (Table S1). The results indicated that the isomers with high symmetry have relatively high stability. However, because the difference in the energies is small (approximately several dozens of millielectronvolts), one can expect that all of these isomers could be synthesized in our experiments. The weaker molecular orbital interactions between metals in TNCs compared with those in Au_*n*_(SR)_*m*_ and Ag_*n*_(SR)_*m*_, in which the orbitals are highly correlated with each other, probably lead to these smaller stability differences between the isomers.^53,54^ It is also presumed that the peaks (**iv**–**xi**) separated by RP-HPLC contain several isomers. To avoid complicating the discussion, we performed the calculations by using each of these isomers with high symmetry (isomers C–I in Table S1) in the following discussion.

We compared the geometric structure of Ni_*x*_Pt_6–*x*_(MT)_12_ (*x* = 0–6) (Figure 3 and Tables S2–S8). A comparison of the monometallic TNCs [Ni_6_(MT)_12_ and Pt_6_(MT)_12_] indicated that the average M–S bond lengths are ∼2.27 and ∼2.40 Å, respectively, and the average S–M–S bond angles are ∼97.7° and ∼99.0°, respectively. Considering the fact that the average Ni–S bond lengths and average S–Ni–S bond angles do not change substantially depending on the ring sizes of Ni_*n*_(SR)_2*n*_ (*n* = 4–6),^18,36^ one can consider that there are appropriate values in these M–S bond lengths and S–M–S bond angles depending on metal element. Next, we compared the average M–M distances (Table S2) and average M–S–M bond angles (Table S3) for Ni_*x*_Pt_6–*x*_(MT)_12_ (*x* = 0–6); these M–M distances (3.02–3.28 Å) and M–S–M bond angles (83.1–86.2°) increased slightly with an increase in the number of Pt atoms. Thus, the overall M–S–M bond angle extends in the equatorial direction of the ring structure with an increase in the number of Pt atoms. For instance, in Ni_2_Pt_4_(MT)_12_, the average Pt–S bond length (2.39 Å) and average S–Pt–S bond angle (99.6°) are longer or wider than the average Ni–S bond length (2.29 Å) and average S–Ni–S bond angle (96.3°), respectively (Figure S14 and Tables S5 and S8). We also observed such a trend for Ni_*x*_Pt_6–*x*_(MT)_12_ (*x* = 1 and 3–5) (Figure S14 and Tables S4–S8). Thus, in Ni_*x*_Pt_6–*x*_(MT)_12_ (*x* = 1–5), the metal-specific M–S bond lengths and S–M–S bond angles are difficult to maintain, resulting in distortions in the ring structure. We also compared the standard deviation (SD) of each bond length and angle to represent the distortion in the ring structure (Figure 3b,c). The SDs of the average M–M–M, S–M–S, and M–S–M bond angles of Ni_*x*_Pt_6–*x*_(MT)_12_ (*x* = 1–5) were larger than those of Ni_6_(MT)_12_ and Pt_6_(MT)_12_ composed of pure metals. Thus, the geometric structure of Ni_*x*_Pt_6–*x*_(MT)_12_ (*x* = 1–5) is more distorted than those of Ni_6_(MT)_12_ and Pt_6_(MT)_12_. In particular, Ni_*x*_Pt_6–*x*_(MT)_12_ (*x* = 1 and 2) has a large distortion, presumably causing its relatively low stability in alloy TNCs (Figure 3c). Probably because of this relatively low stability, metal exchange between TNCs could have easily proceeded in solution. The fact that we produced Ni_*x*_Pt_6–*x*_(MT)_12_, which has a large number of Pt substitutions (i.e., the product with a long retention time), in a small quantity is also qualitatively consistent with the structural stability results (Figure S7B).

**Figure 3:**
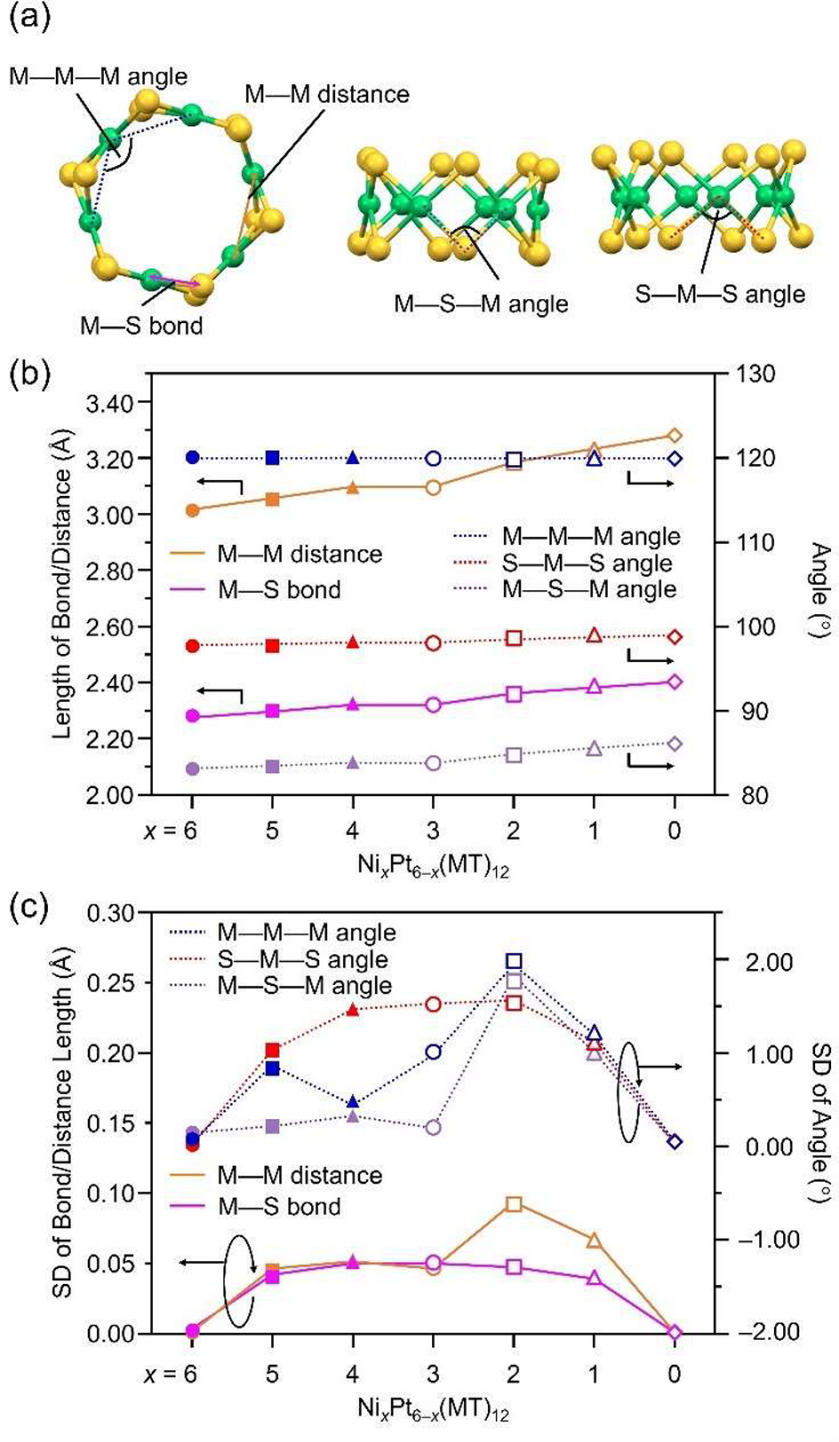
(a) Relevant geometrical parameters for analysis of the structure for Ni_*x*_Pt_6–*x*_(MT)_12_ determined by DFT calculations. (b) Averages and (c) standard deviations (SDs) of M–S bonds as well as M–M distances (angstroms) and M–M–M, S–M–S, and M–S–M angles (degrees).

We then calculated their absorption spectra from the optimized structural model. For Ni_6_(MT)_12_, we observed several peaks corresponding to charge transfer transitions between orbitals centered on the S and Ni atoms in the calculated absorption spectrum (Figure 4). The peaks at ∼340 and ∼420 nm are mainly attributable to the S pσ → Ni d transition, whereas the peak at 550 nm is mainly attributable to the S pπ → Ni d transition.^55^ Further attention to the peak at ∼420 nm indicates that it is mainly attributable to HOMO–*X* (0 ≤ *X* ≤ 1) to LUMO*+X* (0 ≤ *X* ≤ 4) (Figure 5 and Table S9). The HOMO–*X* (0 ≤ *X* ≤ 1) is mainly composed of contributions from the 3p orbital of the S atom, with little contribution from the 3d orbital of the Ni atom, whereas the LUMO*+X* (0 ≤ *X* ≤ 1) is mainly composed of S and Ni.^21^ We investigated the changes in these transitions at ∼420 nm with an increasing number of Pt substitutions in Ni_*x*_Pt_6–*x*_(MT)_12_ (Figure 5 and Figures S15–S21). As the number of Pt substitutions increased [Ni_*x*_Pt_6–*x*_(MT)_12_ (*x* = 0–3)], the transition energy tended to shift toward the lower-energy side (Figure 4), mainly because of an increase in the energy level of the HOMO orbitals. In Ni_3_Pt_3_(MT)_12_, symmetric Pt doping also induced degeneracy in the HOMO to LUMO*+X* (1 ≤ *X* ≤ 2) orbitals similar to those of Ni_6_(MT)_12_ and Pt_6_(MT)_12_. When we further increased the quantity of Pt doping [Ni_*x*_Pt_6–*x*_(MT)_12_ (*x* = 4–6)], the transition attributable to LUMO*+*2 (mainly composed of S and Pt) was newly enhanced at shorter wavelengths [∼400 nm (Figure 4)], and the overall absorption peak was shifted to the shorter wavelength side. Such shifts in absorption peaks at ∼420 nm of each Ni_*x*_Pt_6–*x*_(PET)_12_ (*x* = 0–6) were actually observed in the optical absorption spectra of the fractions separated by RP-HPLC (Figure S7C). However, a clear correlation with calculated results could not be observed because the absorption intensities were not sufficiently high due to the low yield of some Ni_*x*_Pt_6–*x*_(PET)_12_.

**Figure 4:**
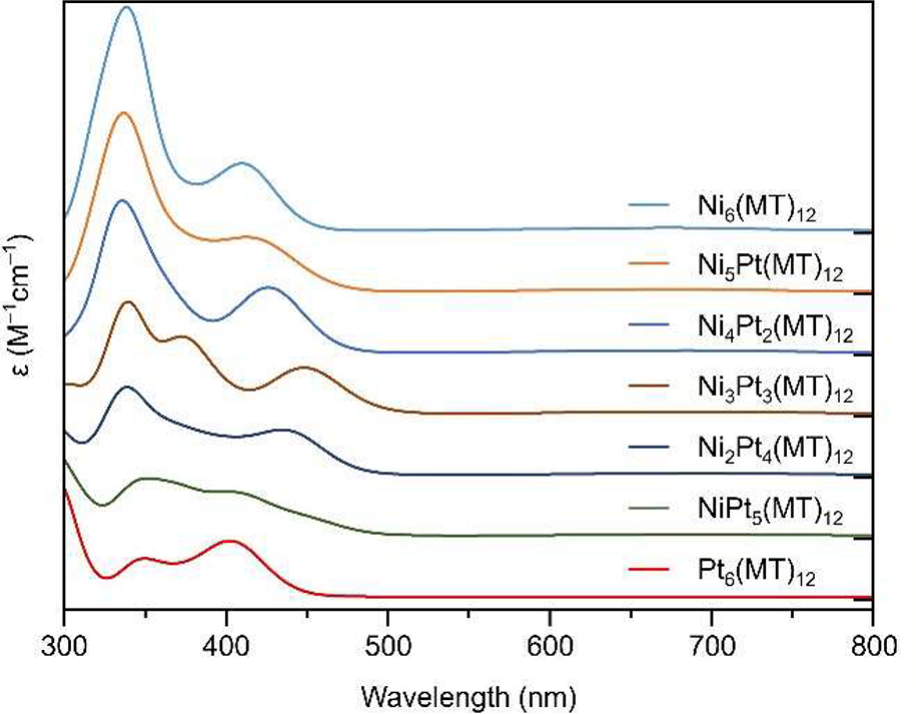
Calculated absorption spectra of [Ni_*x*_Pt_6–*x*_(MT)_12_]^0^ (*x* = 0–6).

**Figure 5:**
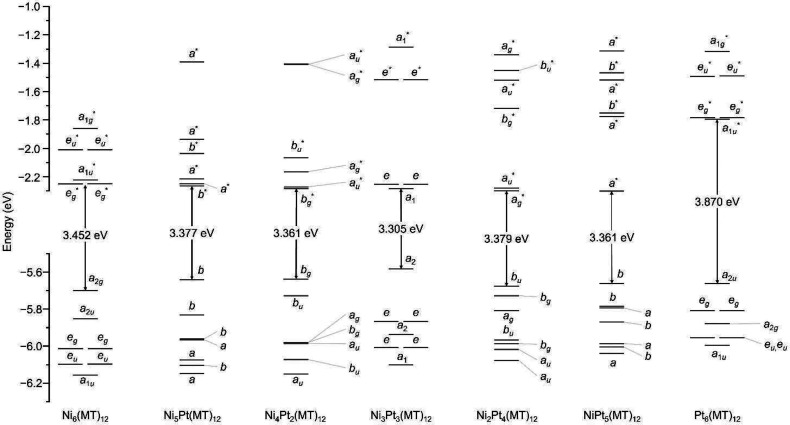
Calculated energy diagram of [Ni_*x*_Pt_6–*x*_(MT)_12_]^0^ (*x* = 0–6).

In conclusion, we synthesized SR-protected hexameric NiPt alloy TNCs and separated them by RP-HPLC. TNCs composed of a single metal can be isolated, but these alloy TNCs [Ni_*x*_Pt_6–*x*_(SR)_12_, where *x* = 1–5] are difficult to isolate because of metal exchange in solution. We further evaluated the electronic structures of Ni_*x*_Pt_6–*x*_(SR)_12_ by DFT calculations. A shift in the absorption wavelength at ∼420 nm is caused by a change in the proportion of Ni and Pt as well as a change in the composed orbital in the HOMOs and LUMOs, which are mainly composed of S and Ni or Pt. The stability of alloy TNCs is lower than that of monometallic TNCs because of distortions in the geometric structure, causing difficulty in isolating alloy TNCs. These findings are important for obtaining guidelines for synthesizing and isolating other types of alloy TNCs.

## Supporting Information Available

Chemicals, experimental and computational details, calculation of vertical transitions, analysis of structural data, MALDI MS data, XAFS spectra, RP-HPLC chromatograms, and optical absorbance spectra (PDF)

## References

[ref1] Kawawaki T.; Ebina A.; Hosokawa Y.; Ozaki S.; Suzuki D.; Hossain S.; Negishi Y. Thiolate-Protected Metal Nanoclusters: Recent Development in Synthesis, Understanding of Reaction, and Application in Energy and Environmental Field. Small 2021, 17, 2005328 10.1002/smll.202005328. 33522090

[ref2] Hossain S.; Hirayama D.; Ikeda A.; Ishimi M.; Funaki S.; Samanta A.; Kawawaki T.; Negishi Y. Atomically Precise Thiolate-Protected Gold Nanoclusters: Current Status of Designability of the Structure and Physicochemical Properties. Aggregate 2023, 4, e255 10.1002/agt2.255.

[ref3] Kawawaki T.; Imai Y.; Suzuki D.; Kato S.; Kobayashi I.; Suzuki T.; Kaneko R.; Hossain S.; Negishi Y. Atomically Precise Alloy Nanoclusters. Chem. - Eur. J. 2020, 26, 16150–16193. 10.1002/chem.202001877. 32453462

[ref4] Chakraborty I.; Pradeep T. Atomically Precise Clusters of Noble Metals: Emerging Link between Atoms and Nanoparticles. Chem. Rev. 2017, 117, 8208–8271. 10.1021/acs.chemrev.6b00769. 28586213

[ref5] Yan J.; Teo B. K.; Zheng N. Surface Chemistry of Atomically Precise Coinage-Metal Nanoclusters: From Structural Control to Surface Reactivity and Catalysis. Acc. Chem. Res. 2018, 51, 3084–3093. 10.1021/acs.accounts.8b00371. 30433756

[ref6] Zhang Q.-F.; Chen X.; Wang L.-S. Toward Solution Syntheses of the Tetrahedral Au_20_ Pyramid and Atomically Precise Gold Nanoclusters with Uncoordinated Sites. Acc. Chem. Res. 2018, 51, 2159–2168. 10.1021/acs.accounts.8b00257. 30070827

[ref7] Yao Q.; Chen T.; Yuan X.; Xie J. Toward Total Synthesis of Thiolate-Protected Metal Nanoclusters. Acc. Chem. Res. 2018, 51, 1338–1348. 10.1021/acs.accounts.8b00065. 29792422

[ref8] Lei Z.; Wan X.-K.; Yuan S.-F.; Guan Z.-J.; Wang Q.-M. Alkynyl Approach toward the Protection of Metal Nanoclusters. Acc. Chem. Res. 2018, 51, 2465–2474. 10.1021/acs.accounts.8b00359. 30272944

[ref9] Gan Z.; Xia N.; Wu Z. Discovery, Mechanism, and Application of Antigalvanic Reaction. Acc. Chem. Res. 2018, 51, 2774–2783. 10.1021/acs.accounts.8b00374. 30379057

[ref10] Nieto-Ortega B.; Bürgi T. Vibrational Properties of Thiolate-Protected Gold Nanoclusters. Acc. Chem. Res. 2018, 51, 2811–2819. 10.1021/acs.accounts.8b00376. 30398341

[ref11] Kwak K.; Lee D. Electrochemistry of Atomically Precise Metal Nanoclusters. Acc. Chem. Res. 2019, 52, 12–22. 10.1021/acs.accounts.8b00379. 30500153

[ref12] Pei Y.; Wang P.; Ma Z.; Xiong L. Growth-Rule-Guided Structural Exploration of Thiolate-Protected Gold Nanoclusters. Acc. Chem. Res. 2019, 52, 23–33. 10.1021/acs.accounts.8b00385. 30548076

[ref13] Agrachev M.; Ruzzi M.; Venzo A.; Maran F. Nuclear and Electron Magnetic Resonance Spectroscopies of Atomically Precise Gold Nanoclusters. Acc. Chem. Res. 2019, 52, 44–52. 10.1021/acs.accounts.8b00495. 30480998

[ref14] Sakthivel N. A.; Dass A. Aromatic Thiolate-Protected Series of Gold Nanomolecules and a Contrary Structural Trend in Size Evolution. Acc. Chem. Res. 2018, 51, 1774–1783. 10.1021/acs.accounts.8b00150. 30027733

[ref15] Konishi K.; Iwasaki M.; Shichibu Y. Phosphine-Ligated Gold Clusters with Core+exo Geometries: Unique Properties and Interactions at the Ligand–Cluster Interface. Acc. Chem. Res. 2018, 51, 3125–3133. 10.1021/acs.accounts.8b00477. 30427180

[ref16] Aikens C. M. Electronic and Geometric Structure, Optical Properties, and Excited State Behavior in Atomically Precise Thiolate-Stabilized Noble Metal Nanoclusters. Acc. Chem. Res. 2018, 51, 3065–3073. 10.1021/acs.accounts.8b00364. 30444598

[ref17] Tang Q.; Hu G.; Fung V.; Jiang D.-e. Insights into Interfaces, Stability, Electronic Properties, and Catalytic Activities of Atomically Precise Metal Nanoclusters from First Principles. Acc. Chem. Res. 2018, 51, 2793–2802. 10.1021/acs.accounts.8b00380. 30398051

[ref18] Tan C.; Jin M.; Ma X.; Zhu Q.; Huang Y.; Wang Y.; Hu S.; Sheng T.; Wu X. *In Situ* Synthesis of Nickel Tiara-Like Clusters with Two Different Thiolate Bridges. Dalton Trans. 2012, 41, 8472–8476. 10.1039/c2dt30524k. 22653469

[ref19] Zhang C.; Matsumoto T.; Samoc M.; Petrie S.; Meng S.; Christopher Corkery T.; Stranger R.; Zhang J.; Humphrey M. G.; Tatsumi K. Dodecanuclear-Ellipse and Decanuclear-Wheel Nickel(II) Thiolato Clusters with Efficient Femtosecond Nonlinear Absorption. Angew. Chem., Int. Ed. 2010, 49, 4209–4212. 10.1002/anie.200907074. 20422664

[ref20] Zhang C.; Takada S.; Kölzer M.; Matsumoto T.; Tatsumi K. Nickel(II) Thiolate Complexes with a Flexible *Cyclo*-{Ni_10_S_20_} Framework. Angew. Chem., Int. Ed. 2006, 45, 3768–3772. 10.1002/anie.200600319. 16622894

[ref21] Datta A.; John N. S.; Kulkarni G. U.; Pati S. K. Aromaticity in Stable Tiara Nickel Thiolates: Computational and Structural Analysis. J. Phys. Chem. A 2005, 109, 11647–11649. 10.1021/jp055344w. 16366612

[ref22] Pan Y.; Chen J.; Gong S.; Wang Z. Co-Synthesis of Atomically Precise Nickel Nanoclusters and the Pseudo-Optical Gap of Ni_4_(SR)_8_. Dalton Trans. 2018, 47, 11097–11103. 10.1039/C8DT02059K. 30040107

[ref23] Kriege M.; Henkel G. [Ni_5_(SEt)_10_] und [Ni_4_(SC_6_H_11_)_8_], Homoleptische Nickel-Thiolate mit Pentagonal-Prismatischen und Cubanartigen Schwefelgerüsten /[Ni_5_(SEt)_10_] and [Ni_4_(SC_6_H_11_)_8_], Homoleptic Nickel-Thiolates with Pentagonal-Prismatic and Cubane-Like Sulfur Frameworks. Z. Naturforsch., B: J. Chem. Sci. 1987, 42b, 1121–1128. 10.1515/znb-1987-0912.

[ref24] Koo B.-K.; Block E.; Kang H.; Liu S.; Zubieta J. Synthesis and Structural Characterization of *Cyclo*-Pentakis [Bis(μ-Trimethylsilylthiomethane)Nickel(II)], [Ni(SCH_2_SiMe_3_)_2_]_5_, a Pentametallic Tiara Structure. Polyhedron 1988, 7, 1397–1399. 10.1016/S0277-5387(00)80392-7.

[ref25] Chen J.; Liu L.; Weng L.; Lin Y.; Liao L.; Wang C.; Yang J.; Wu Z. Synthesis and Properties Evolution of a Family of Tiara-Like Phenylethanethiolated Palladium Nanoclusters. Sci. Rep. 2015, 5, 16628 10.1038/srep16628. 26567806 PMC4644969

[ref26] Chen J.; Pan Y.; Wang Z.; Zhao P. The Fluorescence Properties of Tiara Like Structural Thiolated Palladium Clusters. Dalton Trans. 2017, 46, 12964–12970. 10.1039/C7DT02836A. 28932839

[ref27] Gao X.; Chen W. Highly Stable and Efficient Pd_6_(SR)_12_ Cluster Catalysts for the Hydrogen and Oxygen Evolution Reactions. Chem. Commun. 2017, 53, 9733–9736. 10.1039/C7CC04787H. 28812066

[ref28] Chen J.; Liu L.; Liu X.; Liao L.; Zhuang S.; Zhou S.; Yang J.; Wu Z. Gold-Doping of Double-Crown Pd Nanoclusters. Chem. - Eur. J. 2017, 23, 18187–18192. 10.1002/chem.201704413. 29034569

[ref29] Yang Z.; Smetana A. B.; Sorensen C. M.; Klabunde K. J. Synthesis and Characterization of a New Tiara Pd(II) Thiolate Complex, [Pd(SC_12_H_25_)_2_]_6_, and Its Solution-Phase Thermolysis to Prepare Nearly Monodisperse Palladium Sulfide Nanoparticles. Inorg. Chem. 2007, 46, 2427–2431. 10.1021/ic061242o. 17335274

[ref30] Yamashina Y.; Kataoka Y.; Ura Y. Tiara-Like Octanuclear Palladium(II) and Platinum(II) Thiolates and Their Inclusion Complexes with Dihalo- or Iodoalkanes. Inorg. Chem. 2014, 53, 3558–3567. 10.1021/ic403050c. 24661117

[ref31] Chui S. S.-Y.; Low K.-H.; Lu J.; Roy V. A. L.; Chan S. L.-F.; Che C.-M. Homoleptic Platinum(II) and Palladium(II) Organothiolates and Phenylselenolates: Solvothermal Synthesis, Structural Determination, Optical Properties, and Single-Source Precursors for PdSe and PdS Nanocrystals. Chem. - Asian J. 2010, 5, 2062–2074. 10.1002/asia.201000233. 20665775

[ref32] Imaoka T.; Akanuma Y.; Haruta N.; Tsuchiya S.; Ishihara K.; Okayasu T.; Chun W.-J.; Takahashi M.; Yamamoto K. Platinum Clusters with Precise Numbers of Atoms for Preparative-Scale Catalysis. Nat. Commun. 2017, 8, 688 10.1038/s41467-017-00800-4. 28947792 PMC5613004

[ref33] George A.; Asha K. S.; Reber A. C.; Biltek S. R.; Pedicini A. F.; Sen A.; Khanna S. N.; Mandal S. Atom Precise Platinum-Thiol Crowns. Nanoscale 2015, 7, 19448–19452. 10.1039/C5NR05325K. 26486562

[ref34] Hu W.; Sun Y.; Li S.; Cheng X.; Cai X.; Chen M.; Zhu Y. Visible-Light-Driven Methane Conversion with Oxygen Enabled by Atomically Precise Nickel Catalyst. CCS Chem. 2020, 2, 2509–2519. 10.31635/ccschem.020.202000521.

[ref35] Kauffman D. R.; Alfonso D.; Tafen D. N.; Lekse J.; Wang C.; Deng X.; Lee J.; Jang H.; Lee J.-s.; Kumar S.; Matranga C. Electrocatalytic Oxygen Evolution with an Atomically Precise Nickel Catalyst. ACS Catal. 2016, 6, 1225–1234. 10.1021/acscatal.5b02633.

[ref36] Funaki S.; Kawawaki T.; Okada T.; Takemae K.; Hossain S.; Niihori Y.; Naito T.; Takagi M.; Shimazaki T.; Kikkawa S.; Yamazoe S.; Tachikawa M.; Negishi Y. Improved Activity for the Oxygen Evolution Reaction Using a Tiara-Like Thiolate-Protected Nickel Nanocluster. Nanoscale 2023, 15, 5201–5208. 10.1039/D2NR06952K. 36789780

[ref37] Joya K. S.; Sinatra L.; AbdulHalim L. G.; Joshi C. P.; Hedhili M. N.; Bakr O. M.; Hussain I. Atomically Monodisperse Nickel Nanoclusters as Highly Active Electrocatalysts for Water Oxidation. Nanoscale 2016, 8, 9695–9703. 10.1039/C6NR00709K. 27109550

[ref38] Kagalwala H. N.; Gottlieb E.; Li G.; Li T.; Jin R.; Bernhard S. Photocatalytic Hydrogen Generation System Using a Nickel-Thiolate Hexameric Cluster. Inorg. Chem. 2013, 52, 9094–9101. 10.1021/ic4013069. 23865570

[ref39] Chai X.; Li T.; Chen M.; Jin R.; Ding W.; Zhu Y. Suppressing the Active Site-Blocking Impact of Ligands of Ni_6_(SR)_12_ Clusters with the Assistance of NH_3_ on Catalytic Hydrogenation of Nitriles. Nanoscale 2018, 10, 19375–19382. 10.1039/C8NR03700K. 30307002

[ref40] Kang X.; Li Y.; Zhu M.; Jin R. Atomically Precise Alloy Nanoclusters: Syntheses, Structures, and Properties. Chem. Soc. Rev. 2020, 49, 6443–6514. 10.1039/C9CS00633H. 32760953

[ref41] Khatun E.; Pradeep T. New Routes for Multicomponent Atomically Precise Metal Nanoclusters. ACS Omega 2021, 6, 1–16. 10.1021/acsomega.0c04832. 33458454 PMC7807469

[ref42] Shichibu Y.; Yoshida K.; Konishi K. Hexanuclear Platinum(II) Thiolate Macrocyclic Host: Charge-Transfer-Driven Inclusion of a Ag^I^ Ion Guest. Inorg. Chem. 2016, 55, 9147–9149. 10.1021/acs.inorgchem.6b01579. 27608203

[ref43] Yamashina Y.; Kataoka Y.; Ura Y. Inclusion of an Iodine Molecule in a Tiara-Like Octanuclear Palladium Thiolate Complex. Eur. J. Inorg. Chem. 2014, 2014, 4073–4078. 10.1002/ejic.201402616. 24661117

[ref44] Ivanov S. A.; Kozee M. A.; Merrill W. A.; Agarwal S.; Dahl L. F. *Cyclo*-[Ni(μ_2_-SPh)_2_]_9_ and *Cyclo*-[Ni(μ_2_-SPh)_2_]_11_: New Oligomeric Types of Toroidal Nickel(II) Thiolates Containing Geometrically Unprecedented 9- and 11-Membered Ring Systems. J. Chem. Soc., Dalton Trans. 2002, 4105–4115. 10.1039/B204273H.

[ref45] Akanuma Y.; Imaoka T.; Sato H.; Yamamoto K. Silver in the Center Enhances Room-Temperature Phosphorescence of a Platinum Sub-Nanocluster by 18 Times. Angew. Chem., Int. Ed. 2021, 60, 4551–4554. 10.1002/anie.202012921. 33200557

[ref46] Negishi Y.; Hashimoto S.; Ebina A.; Hamada K.; Hossain S.; Kawawaki T. Atomic-Level Separation of Thiolate-Protected Metal Clusters. Nanoscale 2020, 12, 8017–8039. 10.1039/D0NR00824A. 32207494

[ref47] Feld H.; Leute A.; Rading D.; Benninghoven A.; Henkel G.; Krüger T.; Krebs B. High Mass Resolution Plasma Desorption and Secondary Ion Mass Spectrometry of Neutral Nickel Thiolate Complexes. Crystal Structure of [Ni_6_(SC_3_H_7_)_12_]. Z. Naturforsch., B: J. Chem. Sci. 1992, 47b, 929–936. 10.1515/znb-1992-0706.

[ref48] Hall M. D.; Daly H. L.; Zhang J. Z.; Zhang M.; Alderden R. A.; Pursche D.; Foran G. J.; Hambley T. W. Quantitative Measurement of the Reduction of Platinum(IV) Complexes Using X-Ray Absorption Near-Edge Spectroscopy (XANES). Metallomics 2012, 4, 568–575. 10.1039/c2mt20053h. 22569908

[ref49] Krishnadas K. R.; Baksi A.; Ghosh A.; Natarajan G.; Pradeep T. Structure-Conserving Spontaneous Transformations between Nanoparticles. Nat. Commun. 2016, 7, 13447 10.1038/ncomms13447. 27830711 PMC5110647

[ref50] Zheng K.; Fung V.; Yuan X.; Jiang D.-e.; Xie J. Real Time Monitoring of the Dynamic Intracluster Diffusion of Single Gold Atoms into Silver Nanoclusters. J. Am. Chem. Soc. 2019, 141, 18977–18983. 10.1021/jacs.9b05776. 31609116

[ref51] Niihori Y.; Hashimoto S.; Koyama Y.; Hossain S.; Kurashige W.; Negishi Y. Dynamic Behavior of Thiolate-Protected Gold-Silver 38-Atom Alloy Clusters in Solution. J. Phys. Chem. C 2019, 123, 13324–13329. 10.1021/acs.jpcc.9b02644.

[ref52] Knoppe S.; Dolamic I.; Bürgi T. Racemization of a Chiral Nanoparticle Evidences the Flexibility of the Gold–Thiolate Interface. J. Am. Chem. Soc. 2012, 134, 13114–13120. 10.1021/ja3053865. 22793992

[ref53] Fernández E. M.; Soler J. M.; Garzón I. L.; Balbás L. C. Trends in the Structure and Bonding of Noble Metal Clusters. Phys. Rev. B 2004, 70, 165403 10.1103/PhysRevB.70.165403.

[ref54] Hossain S.; Miyajima S.; Iwasa T.; Kaneko R.; Sekine T.; Ikeda A.; Kawawaki T.; Taketsugu T.; Negishi Y. [Ag_23_Pd_2_(PPh_3_)_10_Cl_7_]: A New Family of Synthesizable Bi-Icosahedral Superatomic Molecules. J. Chem. Phys. 2021, 155, 024302 10.1063/5.0057005. 34266257

[ref55] Gorelsky S. I.; Basumallick L.; Vura-Weis J.; Sarangi R.; Hodgson K. O.; Hedman B.; Fujisawa K.; Solomon E. I. Spectroscopic and DFT Investigation of [M{HB(3,5-^*i*^Pr_2_pz)_3_}(SC_6_F_5_)] (M = Mn, Fe, Co, Ni, Cu, and Zn) Model Complexes: Periodic Trends in Metal-Thiolate Bonding. Inorg. Chem. 2005, 44, 4947–4960. 10.1021/ic050371m. 15998022 PMC2593087

