# Supplementary material for: Tiara-like Hexanuclear Nickel–Platinum Alloy Nanocluster
Source: J Phys Chem Lett. 2024 Feb 1;15(5):1539–45. doi: 10.1021/acs.jpclett.3c03594 (PMC10860137; doi:10.1021/acs.jpclett.3c03594)
Supplement: Supplementary file 1 — jz3c03594_si_001.pdf [file jz3c03594_si_001.pdf]

Supporting Information

Tiara-like Hexanuclear Nickel–Platinum Alloy Nanocluster

Tomoshige Okada,[1] Tokuhisa Kawawaki,[1, 2, *] Kana Takemae,[1] Shiho Tomihari,[1] Taiga Kosaka,[1] Yoshiki Niihori,[2] and Yuichi Negishi[1, 2, *]

[1]Department of Applied Chemistry, Faculty of Science, Tokyo University of Science, 1−3 Kagurazaka, Shinjuku-ku, Tokyo 162−8601, Japan

[2]Research Institute for Science and Technology, Tokyo University of Science, 2641 Yamazaki, Noda, Chiba 278−8510, Japan

[*]Corresponding author E-mail: kawawaki@rs.tus.ac.jp (T.K.), negishi@rs.tus.ac.jp (Y.N.)

1 Chemicals

All chemicals were commercially obtained and used without further purification. Silver trifluoroacetate, nickel(II) nitrate hexahydrate [$Ni(NO_3)_2 \cdot 6H_2O$], triethylamine (NEt_3), 1-octanethiol, 1-dodecanethiol, Ni standard solution (1000 mg L^{-1}), yttrium standard solution (1000 mg L^{-1}), platinum (Pt) standard solution (1000 mg L^{-1}), bismuth standard solution (1000 mg L^{-1}), Nafion dispersion solution, potassium hydroxide (KOH) and pure water were obtained from FUJIFILM Wako Pure Chemical Co (Japan). Acetone, acetonitrile, 1-propanol, methanol (MeOH), tetrahydrofuran (THF), dichloromethane (DCM), toluene, hydrochloric acid (HCl) and nitric acid (HNO_3) were obtained from Kanto Chemical Co., Inc (Japan), and 2-phenylethanethiol was obtained from Sigma–Aldrich (USA). *trans*-2-[3-(4-*tert*-Butylphenyl)-2-methyl-2-propenylidene]malononitrile (DCTB) was obtained from Tokyo Kasei (Japan). Hydrogen hexachloroplatinate(IV) hexahydrate ($H_2PtCl_6 \cdot 6H_2O$) was purchased from Tanaka Kikinzoku. Carbon black (CB; Vulcan XC-72), dried under vacuum before use, was obtained from Fuel Cell Earth Co., Inc (Japan). Alumina paste (ALUMINA POLISHINNG SUSPENSION) was from Maruto Instrument Co., Ltd. (Tokyo, Japan).

2. Characterization

Matrix-assisted laser desorption/ionization (MALDI) mass spectra were recorded with a JMS-S3000 spiral time-of-flight mass spectrometer (JEOL, Tokyo, Japan) equipped with a semiconductor laser (λ = 349 nm). DCTB was used as the MALDI matrix. To minimize nanocluster (NC) dissociation induced by laser irradiation, the sample-to-matrix ratio was fixed at 1:1000.

The transmission electron microscope (TEM) images were recorded with a H-9500 electron microscope (HITACHI, Tokyo, Japan) or JEM-2100 electron microscope (JEOL, Tokyo, Japan) operating at 200 kV, typically using magnification of 600,000.

Optical absorbance spectra of products were acquired in DCM at 25 °C with a V-630 spectrometer (JASCO, Tokyo, Japan).

Ni K-edge and Pt L_3-edge X-ray absorption fine structure (XAFS) measurements were performed at beamline BL01B1 of the SPring-8 facility of the Japan Synchrotron Radiation Research Institute (proposal numbers 2020A0695, 2021A1102, 2021B1163, 2022A1075, and 2022B1823). The incident X-ray beam was monochromatized with a Si(111) double-crystal monochromator. XAFS spectra of all samples (as well as Ni foil, solid NiO, Pt foil, and solid PtO_2 as a reference) were recorded in transmission mode with ionization chambers. The X-ray energies for the Ni K-edges and Pt L_3-edges were calibrated with Ni foil and Pt foil, respectively. X-ray absorption near-edge structure (XANES) and extended XAFS (EXAFS) spectra were analyzed with xTunes[1] as follows. The χ spectra were extracted by subtracting the atomic absorption background by cubic spline interpolation and normalized to the edge height. The normalized data were used as the XANES spectra. The k^3-weighted χ spectra in the k range 3.0–13.0 Å^{-1} for the Ni K-edge and Pt L_3-edge were Fourier-transformed into r space for structural analysis.

Separation and isolation of products were performed with a Shimadzu Prominence high-performance liquid chromatography (HPLC) system consisting of LC-20AD (2 pumps), an SPD-M20A [photodiode array (PDA) detector], a CTO-20AC (column oven), and a DGU-20A3R (on-line degasser). A YMC core–shell ODS column (Meteoric Core C18, 150 mm × 4.6 mm, I.D., 2.7 μm) was used as the stationary phase. The column and detector were aged (stabilized) for sufficient time prior to analysis. Reversed phase (RP) mode was used for separation. The mobile phase was continuously changed from pure acetonitrile to pure acetone/toluene by the gradient program. The flow rate of the mobile phase was set to 1.0 mL/min, and the optical absorption spectra of the separated products were obtained with a PDA detector in the range of 190–800 nm. The chromatogram was obtained from the absorption intensity at 420 nm.

Inductively coupled plasma-mass spectrometry was performed with an Agilent 7850c spectrometer (Agilent Technologies, Tokyo, Japan). Yttrium was used as the internal standard for Ni and bismuth as the internal standard for Pt.

The Pt 4f and Ni 2p X-ray photoelectron spectroscopy (XPS) spectra were collected by using a JPS-9030 electron spectrometer (JEOL, Tokyo, Japan) at a base pressure of ~2×10^{-8} Torr. X-rays from the Mg-Kα line (1253.6 eV) were used for excitation. Each TNCs was deposited on a molybdenum (Mo) plate and the spectra were calibrated with the peak energies of Mo $3d_{5/2}$ (227.7 eV).

General density functional theory (DFT) and time-dependent (TD)–DFT calculations were performed with Gaussian 16 (ES64L-G16, RevB.01)[2]. To reduce the CPU cost, all 2-phenylethanethiolate (PET) ligands in the cluster were replaced with SCH_3 (MT) ligands. The optimized geometric structure of $Ni_xPt_{6-x}(MT)_{12}$ (x = 0–6) in the ground state was determined by using the BP86 functional[3] with the basis sets of def2-SV(P)[4] for Ni and Pt atoms; and 6-31G(d,p)[5] for H, C, and S atoms. SDD pseudopotentials with scalar relativistic effects were used.[6] Note that no symmetry constraints were added during the calculations. The obtained structure was confirmed to be an optimized structure by harmonic vibrational frequency analysis. Using these optimized structures, vertical transition energies of $S_0 \rightarrow S_n$ (n = 1−100) and corresponding oscillator strengths were calculated by using the TD-B3LYP functional.[7–9] In this calculation, the basis set def2-SV(P) was used for the Ni and Pt atoms; 6-31G(d,p) for the H, C, and S atoms; and the SDD pseudopotential was also used. The molecular geometries and MOs were drawn with Avogadro 1.2.0. The irreducible representation of each molecular orbital was confirmed by recalculating with symmetry constraints.

3. Experiment

Synthesis of **1** [$Ni_xPt_{6-x}(PET)_{12}$ (x = 0–6)]

The overall reaction process was carried out in a 50-mL vial at room temperature and under air. First, $Ni(NO_3)_2·6H_2O$, (100 mg, 0.34 mmol) and 0.2 M $H_2PtCl_6·6H_2O$ (1.7 mL, 0.34 mmol) were added to 1-propanol (12 mL) and stirred for 20 min until complete dissolution. The solution was then stirred vigorously and 2-phenylethanethiol (0.186 mL, 1.38 mmol) was slowly added to the solution. This caused the color of the solution to gradually change from light green to brown. The solution was stirred for 15 min to react with divalent Ni ions (Ni^{2+}), tetravalent platinum ions (Pt^{4+}), and 2-phenylethanethiol. NEt_3 (0.5 mL) was then added to this solution straight away. The color of the solution immediately changed to dark brown. After stirring for 3 h, the crude product was washed with MeOH to remove, for example, unreacted Ni^{2+}, Pt^{4+}, 2-phenylethanethiol, and NEt_3. The crude product was then extracted with DCM (Scheme S1).

Synthesis of **2** [$Ni_xPt_{6-x}(SC_3H_7)_{12}$ (x = 0–6)]

For synthesis of **2**, 2-phenylethanethiol in the synthetic scheme of **1** was replaced with 1-propanethiol in the same mol number.

Synthesis of **3** [$Ni_xPt_{6-x}(SC_8H_{17})_{12}$ (x = 0–6)]

For synthesis of **3**, 2-phenylethanethiol in the synthetic scheme of **1** was replaced with 1-octanethiol in the same mol number.

Synthesis of **4** [$Ni_xPt_{6-x}(SC_{12}H_{25})_{12}$ (x = 0–6)]

For synthesis of **4**, 2-phenylethanethiol in the synthetic scheme of **1** was replaced with 1-dodecanethiol in the same mol number.

Synthesis of **5** [$Ni_xPt_{6-x}(PET)_{12}$ (x = 0–6) via metal exchange reaction]

The overall reaction process was carried out in a 50-mL vial at room temperature and under air. First, $Ni_6(PET)_{12}$ (2.0 mg, 1 μmol), synthesized in accordance with a previous report,[10] was dissolved in 10 mL of THF. H_2PtCl_6 (1 μmol), dissolved in 2 mL of THF, was added to this $Ni_6(PET)_{12}$ solution and stirred for 30 min. Then, the THF solution was removed with a rotary evaporator, and the target material was extracted with DCM. Finally, the insoluble components were removed by filtration to obtain **5**.

Preparation of $Ni_6(PET)_{12}$/CB and Product **1**/CB (1 μmol of metal per 4 mg of CB)

$Ni_6(PET)_{12}$/CB and Product **1**/CB (1 μmol of metal per 4 mg of CB) was prepared by the impregnation method. Each cluster dissolved in THF solution was dropped on the CB and then the catalysts were dried.

Preparation of catalyst slurry

To conduct the electrochemical measurements on $Ni_6(PET)_{12}$/CB and Product **1**/CB (1 μmol of metal per 4 mg of CB), a catalyst slurry was prepared. First, the catalyst powder (10.4 mg; $Ni_6(PET)_{12}$/CB and Product **1**/CB (1 μmol of metal per 4 mg of CB)), was dispersed in a solution containing H_2O (19.1 mL) and 2-propanol (6.0 mL). Then, Nafion™ solution (100 μL) was added to this solution. The vial containing this mixture was sealed and ultrasonicated for 60 min in an ice bath.

Electrochemical measurements

All electrochemical measurements for the hydrogen evolution reaction were performed with an ECstat-302 (EC FRONTIER, Kyoto, Japan) with a RRDE-3A rotating ring disk electrode apparatus (BAS, Tokyo, Japan). A rotating disk electrode (RDE, φ = 5 mm) was polished with alumina paste and then sonicated in water before use. A Pt ring electrode was used as the counter electrode. A silver/silver chloride (Ag/AgCl) electrode was used as the reference electrode. In the setup, first the catalyst slurry was sonicated in an ice bath for 30 min, and then 10 μL of the catalyst slurry was carefully dropped onto the RDE by the drop-cast method. After the catalyst slurry was sufficiently dried, each electrode was set in an electrochemical measurement system containing 0.10 mol L^{-1} KOH (pH = 13) as the electrolyte.

In the measurements, N_2 gas was bubbled for 30 min and then cyclic voltammetry (CV) was conducted 100 times in the region from 0.0 to 1.0 V (vs. reversible hydrogen electrode; RHE) at a scanning rate of 200 mV s^{-1} for cleaning the electrodes. After CV, linear sweep voltammetry (LSV) was performed under N_2 in the region from 0.1 to −0.7 V (vs. RHE) at a rate of 20 mV s^{-1}.

3. Tables

Table S1. Calculated corresponding energies for $Ni_xPt_{6-x}(MT)_{12}$ (x = 0–6)

Composition	Isomer [a]	ΔE (meV) [b]	PG [c]	h [d]	g [e]	P [f]
$Ni_6(MT)_{12}$	–	–	D_{3d}	12	1	–
$Ni_5Pt(MT)_{12}$	–	–	C_2	2	6	–
$Ni_4Pt_2(MT)_{12}$	A (1,2)	15.1	C_2	2	6	0.277
	B (1,3)	0	C_s	2	6	0.497
	C (1,4)	2.56	C_{2h}	4	3	0.225
$Ni_3Pt_3(MT)_{12}$	D (1,2,3)	34.7	C_s	2	6	0.151
	E (1,2,4)	14.7	C_1	1	12	0.656
	F (1,3,5)	0	D_3	6	2	0.193
$Ni_2Pt_4(MT)_{12}$	G (1,2,3,4)	27.4	C_2	2	6	0.217
	H (1,3,4,5)	7.48	C_s	2	6	0.469
	I (1,2,4,5)	0	C_{2h}	4	3	0.313
$NiPt_5(MT)_{12}$	–	–	C_s	2	6	–
$Pt_6(MT)_{12}$	–	–	D_{3d}	12	1	–

[a] Numbers in brackets indicate the position of the substituted Pt atom in the cyclic Ni_6. [b] Energy difference compared with most stable isomer. [c] Point group. [d] Order of point group. [e] Degeneracy of molecule, which was calculated by dividing the order of the point group D_{3d} in $Ni_6(MT)_{12}$ (h = 12) by the order of the point group in the molecule of interest. [f] Boltzmann distribution at 300 K, which was obtained with the following equation: $P_i = g_i \exp(-\Delta E_i/k_B T)/Z$, $Z = \sum g_j \exp(-\Delta E_j/k_B T)$; where ΔE_i is the energy difference between the stable isomer and the target molecule i, k_B is Boltzmann's constant, and T is temperature. Note that the Boltzmann distribution was calculated between isomers with the same composition. It was presumed that various structural isomers can exist because ΔE of $Ni_xPt_{6-x}(MT)_{12}$ (x = 2–4) is substantially small (room temperature ~ 26 meV). For example, in $Ni_4Pt_2(MT)_{12}$, the probability of existence of isomers A, B, and C is approximately 27.7%, 49.7%, and 22.5%, respectively, at 300 K.

Table S2. Average molecular bonds and distances for $Ni_xPt_{6-x}(MT)_{12}$ (x = 0–6)

Sample	M–S (Å)	M–M (Å)	M–center of gravity (Å)
$Ni_6(MT)_{12}$	2.27 ± 0.00	3.02 ± 0.00	3.03 ± 0.06
$Ni_5Pt(MT)_{12}$	2.30 ± 0.04	3.06 ± 0.05	3.02 ± 0.00
$Ni_4Pt_2(MT)_{12}$	2.32 ± 0.05	3.10 ± 0.05	3.06 ± 0.03
$Ni_3Pt_3(MT)_{12}$	2.32 ± 0.05	3.10 ± 0.05	3.10 ± 0.03
$Ni_2Pt_4(MT)_{12}$	2.36 ± 0.05	3.18 ± 0.09	3.14 ± 0.05
$NiPt_5(MT)_{12}$	2.38 ± 0.04	3.23 ± 0.07	3.19 ± 0.05
$Pt_6(MT)_{12}$	2.40 ± 0.00	3.28 ± 0.00	3.23 ± 0.04

Table S3. Average molecular angles for $Ni_xPt_{6-x}(MT)_{12}$ (x = 0–6)

Sample	M–S–M (°)	S–M–S (°)	M–M–M (°)
$Ni_6(MT)_{12}$	83.1 ± 0.14	97.7 ± 0.01	120.0 ± 0.05
$Ni_5Pt(MT)_{12}$	83.4 ± 0.21	97.9 ± 1.03	120.0 ± 0.86
$Ni_4Pt_2(MT)_{12}$	83.8 ± 0.33	98.0 ± 1.47	120.0 ± 0.44
$Ni_3Pt_3(MT)_{12}$	83.8 ± 0.19	98.0 ± 1.52	120.0 ± 0.97
$Ni_2Pt_4(MT)_{12}$	84.8 ± 1.82	98.5 ± 1.56	120.0 ± 1.94
$NiPt_5(MT)_{12}$	85.5 ± 1.00	98.7 ± 1.12	120.0 ± 1.19
$Pt_6(MT)_{12}$	86.2 ± 0.03	99.0 ± 0.02	120.0 ± 0.05

Table S4. Molecular distances of M–M (Å) for $Ni_xPt_{6-x}(MT)_{12}$ (x = 0–6)

$Ni_6(MT)_{12}$			$Ni_5Pt(MT)_{12}$			$Ni_4Pt_2(MT)_{12}$			$Ni_3Pt_3(MT)_{12}$		
**M**	**M**	**length**	**M**	**M**	**length**	**M**	**M**	**length**	**M**	**M**	**length**
Ni	Ni	3.015	Ni	Ni	3.020	Ni	Ni	3.025	Ni	Pt	3.031
Ni	Ni	3.015	Ni	Ni	3.021	Ni	Ni	3.025	Ni	Pt	3.032
Ni	Ni	3.017	Ni	Ni	3.027	Ni	Pt	3.133	Ni	Pt	3.126
Ni	Ni	3.017	Ni	Ni	3.028	Ni	Pt	3.133	Ni	Pt	3.126
Ni	Ni	3.019	Ni	Pt	3.122	Ni	Pt	3.134	Ni	Pt	3.133
Ni	Ni	3.019	Ni	Pt	3.123	Ni	Pt	3.134	Ni	Pt	3.133

$Ni_2Pt_4(MT)_{12}$			$NiPt_5(MT)_{12}$			$Pt_6(MT)_{12}$		
**M**	**M**	**length**	**M**	**M**	**length**	**M**	**M**	**length**
Pt	Ni	3.118	Pt	Ni	3.140	Pt	Pt	3.279
Pt	Ni	3.118	Pt	Ni	3.140	Pt	Pt	3.280
Pt	Ni	3.119	Pt	Pt	3.262	Pt	Pt	3.280
Pt	Ni	3.119	Pt	Pt	3.262	Pt	Pt	3.280
Pt	Pt	3.317	Pt	Pt	3.296	Pt	Pt	3.280
Pt	Pt	3.317	Pt	Pt	3.296	Pt	Pt	3.281

Table S5. Molecular bonds of M–S (Å) for $Ni_xPt_{6-x}(MT)_{12}$ (x = 0–6)

$Ni_6(MT)_{12}$		$Ni_5Pt(MT)_{12}$		$Ni_4Pt_2(MT)_{12}$		$Ni_3Pt_3(MT)_{12}$		$Ni_2Pt_4(MT)_{12}$		$NiPt_5(MT)_{12}$		$Pt_6(MT)_{12}$	
**M**	**length**	**M**	**length**	**M**	**length**	**M**	**length**	**M**	**length**	**M**	**length**	**M**	**length**
Ni	2.271	Ni	2.271	Ni	2.273	Pt	2.272	Ni	2.294	Ni	2.294	Pt	2.400
Ni	2.271	Ni	2.271	Ni	2.273	Ni	2.272	Ni	2.294	Ni	2.294	Pt	2.400
Ni	2.271	Ni	2.271	Ni	2.274	Ni	2.272	Ni	2.294	Ni	2.295	Pt	2.400
Ni	2.271	Ni	2.271	Ni	2.274	Pt	2.273	Ni	2.294	Ni	2.295	Pt	2.400
Ni	2.272	Ni	2.272	Ni	2.277	Pt	2.277	Ni	2.295	Pt	2.384	Pt	2.400
Ni	2.272	Ni	2.272	Ni	2.277	Pt	2.277	Ni	2.295	Pt	2.385	Pt	2.400
Ni	2.272	Ni	2.272	Ni	2.277	Ni	2.277	Ni	2.295	Pt	2.388	Pt	2.400
Ni	2.272	Ni	2.273	Ni	2.277	Ni	2.277	Ni	2.295	Pt	2.388	Pt	2.400
Ni	2.272	Ni	2.277	Ni	2.291	Ni	2.292	Pt	2.385	Pt	2.398	Pt	2.401

Ni	2.272	Ni	2.277	Ni	2.291	Ni	2.292	Pt	2.385	Pt	2.399	Pt	2.401
Ni	2.272	Ni	2.277	Ni	2.291	Ni	2.293	Pt	2.385	Pt	2.400	Pt	2.401
Ni	2.272	Ni	2.277	Ni	2.291	Ni	2.293	Pt	2.385	Pt	2.400	Pt	2.401
Ni	2.277	Ni	2.278	Ni	2.295	Ni	2.294	Pt	2.389	Pt	2.401	Pt	2.401
Ni	2.277	Ni	2.278	Ni	2.295	Ni	2.294	Pt	2.389	Pt	2.401	Pt	2.401
Ni	2.277	Ni	2.278	Ni	2.295	Ni	2.294	Pt	2.389	Pt	2.401	Pt	2.401
Ni	2.277	Ni	2.278	Ni	2.295	Ni	2.295	Pt	2.389	Pt	2.401	Pt	2.401
Ni	2.278	Ni	2.291	Pt	2.386	Pt	2.386	Pt	2.399	Pt	2.401	Pt	2.401
Ni	2.278	Ni	2.292	Pt	2.386	Pt	2.386	Pt	2.399	Pt	2.402	Pt	2.401
Ni	2.278	Ni	2.294	Pt	2.386	Pt	2.386	Pt	2.400	Pt	2.402	Pt	2.401
Ni	2.278	Ni	2.295	Pt	2.387	Pt	2.386	Pt	2.400	Pt	2.402	Pt	2.401
Ni	2.278	Pt	2.386	Pt	2.391	Pt	2.391	Pt	2.403	Pt	2.402	Pt	2.401
Ni	2.278	Pt	2.386	Pt	2.391	Pt	2.391	Pt	2.403	Pt	2.402	Pt	2.402
Ni	2.279	Pt	2.391	Pt	2.392	Pt	2.392	Pt	2.403	Pt	2.404	Pt	2.402
Ni	2.279	Pt	2.392	Pt	2.392	Pt	2.392	Pt	2.403	Pt	2.405	Pt	2.402

Table S6. Molecular angles of M–S–M (°) for $Ni_xPt_{6-x}(MT)_{12}$ (x = 0–6)

$Ni_6(MT)_{12}$			$Ni_5Pt(MT)_{12}$			$Ni_4Pt_2(MT)_{12}$			$Ni_3Pt_3(MT)_{12}$		
**M**	**M**	**angle**	**M**	**M**	**angle**	**M**	**M**	**angle**	**M**	**M**	**angle**
Ni	Ni	82.91	Ni	Ni	83.08	Ni	Ni	83.25	Ni	Pt	83.45
Ni	Ni	82.91	Ni	Ni	83.10	Ni	Ni	83.25	Ni	Pt	83.47
Ni	Ni	82.96	Ni	Ni	83.30	Ni	Ni	83.41	Ni	Pt	83.66
Ni	Ni	82.96	Ni	Ni	83.30	Ni	Ni	83.41	Ni	Pt	83.66
Ni	Ni	82.99	Ni	Ni	83.30	Ni	Pt	83.88	Ni	Pt	83.69
Ni	Ni	82.99	Ni	Ni	83.31	Ni	Pt	83.90	Ni	Pt	83.69
Ni	Ni	83.16	Ni	Pt	83.54	Ni	Pt	83.90	Ni	Pt	83.80
Ni	Ni	83.16	Ni	Pt	83.54	Ni	Pt	83.91	Ni	Pt	83.80
Ni	Ni	83.22	Ni	Ni	83.60	Ni	Pt	84.07	Ni	Pt	83.90
Ni	Ni	83.22	Ni	Ni	83.61	Ni	Pt	84.08	Ni	Pt	83.90
Ni	Ni	83.29	Ni	Pt	83.71	Ni	Pt	84.11	Ni	Pt	84.07
Ni	Ni	83.29	Ni	Pt	83.72	Ni	Pt	84.11	Ni	Pt	84.08

$Ni_2Pt_4(MT)_{12}$			$NiPt_5(MT)_{12}$			$Pt_6(MT)_{12}$		
**M**	**M**	**angle**	**M**	**M**	**angle**	**M**	**M**	**angle**
Ni	Pt	83.46	Ni	Pt	84.19	Pt	Pt	86.13
Ni	Pt	83.46	Ni	Pt	84.19	Pt	Pt	86.15
Ni	Pt	83.46	Ni	Pt	84.29	Pt	Pt	86.16
Ni	Pt	83.46	Ni	Pt	84.29	Pt	Pt	86.16
Ni	Pt	83.56	Pt	Pt	85.55	Pt	Pt	86.16
Ni	Pt	83.56	Pt	Pt	85.56	Pt	Pt	86.17
Ni	Pt	83.56	Pt	Pt	85.58	Pt	Pt	86.18
Ni	Pt	83.56	Pt	Pt	85.58	Pt	Pt	86.19
Pt	Pt	87.29	Pt	Pt	86.63	Pt	Pt	86.20
Pt	Pt	87.29	Pt	Pt	86.64	Pt	Pt	86.20
Pt	Pt	87.45	Pt	Pt	86.73	Pt	Pt	86.21
Pt	Pt	87.45	Pt	Pt	86.73	Pt	Pt	86.22

Table S7. Molecular angles of M–M–M (°) for $Ni_xPt_{6-x}(MT)_{12}$ (x = 0–6)

$Ni_6(MT)_{12}$				$Ni_5Pt(MT)_{12}$				$Ni_4Pt_2(MT)_{12}$			
**M**	**M**	**M**	**angle**	**M**	**M**	**M**	**angle**	**M**	**M**	**M**	**angle**
Ni	Ni	Ni	119.95	Ni	Ni	Pt	118.92	Ni	Ni	Pt	119.67
Ni	Ni	Ni	119.95	Ni	Ni	Pt	118.94	Ni	Ni	Pt	119.68
Ni	Ni	Ni	119.99	Ni	Pt	Ni	119.73	Ni	Ni	Pt	119.70
Ni	Ni	Ni	119.99	Ni	Ni	Ni	120.63	Ni	Ni	Pt	119.72
Ni	Ni	Ni	120.06	Ni	Ni	Ni	120.66	Ni	Pt	Ni	120.60
Ni	Ni	Ni	120.06	Ni	Ni	Ni	121.12	Ni	Pt	Ni	120.64

$Ni_3Pt_3(MT)_{12}$				$Ni_2Pt_4(MT)_{12}$				$NiPt_5(MT)_{12}$			
**M**	**M**	**M**	**angle**	**M**	**M**	**M**	**angle**	**M**	**M**	**M**	**angle**
Pt	Ni	Pt	118.06	Pt	Ni	Pt	117.25	Pt	Pt	Pt	117.86
Pt	Ni	Pt	120.01	Pt	Ni	Pt	117.25	Pt	Pt	Pt	119.74
Pt	Ni	Pt	120.04	Ni	Pt	Pt	121.37	Pt	Pt	Pt	119.76
Ni	Pt	Ni	120.27	Ni	Pt	Pt	121.37	Pt	Ni	Pt	119.90

Ni	Pt	Ni	120.30	Ni	Pt	Pt	121.38	Ni	Pt	Pt	121.36
Ni	Pt	Ni	121.32	Ni	Pt	Pt	121.38	Ni	Pt	Pt	121.38

**$Pt_6(MT)_{12}$**			
**M**	**M**	**M**	**angle**
Pt	Pt	Pt	119.92
Pt	Pt	Pt	119.97
Pt	Pt	Pt	119.98
Pt	Pt	Pt	120.01
Pt	Pt	Pt	120.04
Pt	Pt	Pt	120.08

Table S8. Molecular angles of S–M–S (°) for $Ni_xPt_{6-x}(MT)_{12}$ (x = 0–6)

**$Ni_6(MT)_{12}$**		**$Ni_5Pt(MT)_{12}$**		**$Ni_4Pt_2(MT)_{12}$**		**$Ni_3Pt_3(MT)_{12}$**		**$Ni_2Pt_4(MT)_{12}$**		**$NiPt_5(MT)_{12}$**		**$Pt_6(MT)_{12}$**	
**M**	**angle**	**M**	**angle**	**M**	**angle**	**M**	**angle**	**M**	**angle**	**M**	**angle**	**M**	**angle**
Ni	97.72	Ni	96.76	Ni	96.75	Ni	96.12	Ni	96.29	Ni	96.3	Pt	98.91
Ni	97.72	Ni	96.76	Ni	96.77	Ni	96.14	Ni	96.29	Ni	96.32	Pt	98.93
Ni	97.74	Ni	97.12	Ni	96.77	Ni	96.80	Ni	96.29	Pt	98.91	Pt	98.95
Ni	97.74	Ni	97.13	Ni	96.78	Ni	96.81	Ni	96.29	Pt	98.91	Pt	98.95
Ni	97.75	Ni	97.78	Ni	97.24	Ni	97.23	Pt	99.23	Pt	98.92	Pt	98.96
Ni	97.75	Ni	97.79	Ni	97.24	Ni	97.23	Pt	99.23	Pt	98.92	Pt	98.96
Ni	97.75	Ni	97.84	Ni	97.24	Pt	97.89	Pt	99.23	Pt	98.98	Pt	98.96
Ni	97.75	Ni	97.84	Ni	97.24	Pt	97.90	Pt	99.23	Pt	98.99	Pt	98.96
Ni	97.75	Ni	97.85	Pt	100.08	Pt	100.04	Pt	99.87	Pt	99.33	Pt	98.98
Ni	97.75	Ni	97.85	Pt	100.08	Pt	100.05	Pt	99.87	Pt	99.34	Pt	98.98
Ni	97.76	Pt	100.00	Pt	100.08	Pt	100.05	Pt	99.87	Pt	99.8	Pt	98.99
Ni	97.76	Pt	100.01	Pt	100.10	Pt	100.06	Pt	99.87	Pt	99.81	Pt	98.99

Table S9. Characteristic vertical transition wavelengths (energies), oscillator strengths (*f*), configuration interactions (CI), and expansion coefficients (P) of $Ni_xPt_{6-x}(MT)_{12}$ (x = 0–6) obtained by theoretical calculations.

Molecule	Excited state	Wavelength / nm (eV) [a]	*f*	Dominant configuration	CI expansion coefficient [b]
$Ni_6(MT)_{12}$	S_{28}	413.86 (2.9958)	0.0563	H–1(a_{2u}) → L(e_g^*)	0.41182 (33%)
				H(a_{2g}) → L+4(e_u^*)	0.44250 (39%)
	S_{29}	413.81 (2.9961)	0.0562	H–1(a_{2u}) →L+1(e_g^*)	−0.40975 (33%)
				H(a_{2g}) → L+3(e_u^*)	0.44432 (39%)
	S_{30}	408.68 (0.0171)	0.0171	H(a_{2g}) → L+2(a_{1u}^*)	0.58425 (68%)
$Ni_5Pt(MT)_{12}$	S_{22}	435.08 (2.8497)	0.0248	H(b) → L(b^*)	0.59355 (70%)
	S_{23}	428.27 (2.8950)	0.0120	H(b) → L+1(a^*)	0.55308 (61%)
				H(b) → L+2(a^*)	0.27812 (15%)
	S_{24}	418.92 (2.9596)	0.0231	H(b) → L+3(b^*)	0.50907 (51%)
	S_{25}	417.48 (2.9698)	0.0307	H–2(b) → L+1(a^*)	−0.30200 (18%)
				H(b) → L+4(a^*)	0.33220 (22%)
	S_{26}	407.29 (3.0441)	0.0187	H–1(b) → L(b^*)	0.52000 (54%)
$Ni_4Pt_2(MT)_{12}$	S_{19}	432.69 (2.8654)	0.0338	H(b_g) → L+1(a_u^*)	0.63767 (81%)
	S_{20}	431.34 (2.8744)	0.0657	H–1(b_u) → L(b_g^*)	0.47725 (45%)
				H(b_g) → L+3(b_u^*)	−0.41365 (34%)
	S_{22}	418.89 (2.9598)	0.0044	H–1(b_u) → L(b_g^*)	0.41200 (33%)
				H(b_g) → L+3(b_u^*)	0.43940 (38%)
	S_{23}	418.09 (2.9655)	0.0438	H–1(b_u) → L+2(a_g^*)	0.58963 (69%)
$Ni_3Pt_3(MT)_{12}$	S_{13}	465.94 (2.6609)	0.0118	H(a_2) → L(a_1)	0.62565 (78%)
	S_{14}	447.51 (2.7706)	0.0459	H(a_2) → L+1(e)	0.64358 (82%)
	S_{15}	447.51 (2.7706)	0.0459	H(a_2) → L+2(e)	0.64358 (82%)
$Ni_2Pt_4(MT)_{12}$	S_{10}	440.82 (2.8126)	0.0835	H–1(b_g) → L+1(a_u^*)	−0.42456 (36%)
				H(b_u) → L(a_g^*)	0.48950 (47%)
$NiPt_5(MT)_{12}$	S_5	449.40 (2.7589)	0.0326	H–1(b) → L(a^*)	−0.32821 (21%)
				H(b) → L(a^*)	0.55024 (60%)
	S_8	415.51 (2.9839)	0.0192	H–2(a) → L+1(a^*)	0.41105 (33%)
	S_9	415.50 (2.9839)	0.0215	H–3(b) → L+1(a^*)	0.28298 (16%)
				H–1(b) → L+1(a^*)	0.29589 (17%)
				H(b) → L+1(a^*)	0.31027 (19%)
	S_{14}	401.19 (3.0904)	0.0342	H(b) → L+2(b^*)	0.03420 (32%)
$Pt_6(MT)_{12}$	S_2	414.13 (2.9939)	0.0243	H–6(a_{1u}) → L+1(e_g^*)	−0.29765 (17%)
				H–1(e_g) → L(a_{1u}^*)	0.44926 (40%)
	S_3	414.06 (2.9944)	0.0246	H–6(a_{1u}) → L+2(e_g^*)	0.29877 (17%)
				H–2(e_g) → L(a_{1u}^*)	0.44899 (40%)
	S_9	399.78 (3.1013)	0.0369	H(a_{2u}) → L+1(e_g^*)	0.36503 (26%)
	S_{10}	399.74 (3.1016)	0.0373	H(a_{2u}) → L+2(e_g^*)	0.36611 (26%)

[a]Values in brackets are in electron volts. [b]Values in brackets are the percentage contribution ($P = 2|CI|^2$).

4. Additional schemes

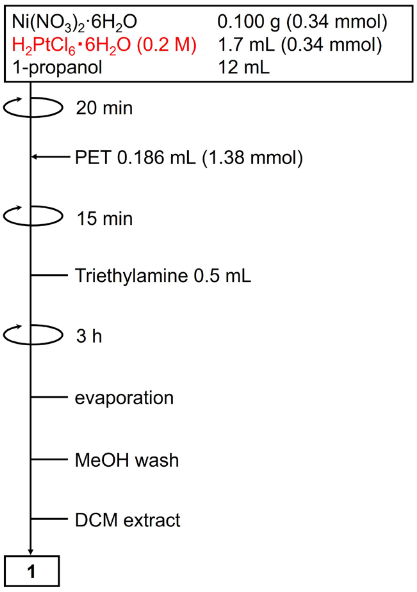

Scheme S1. Synthesis protocol used for **1**.

5. Additional figures

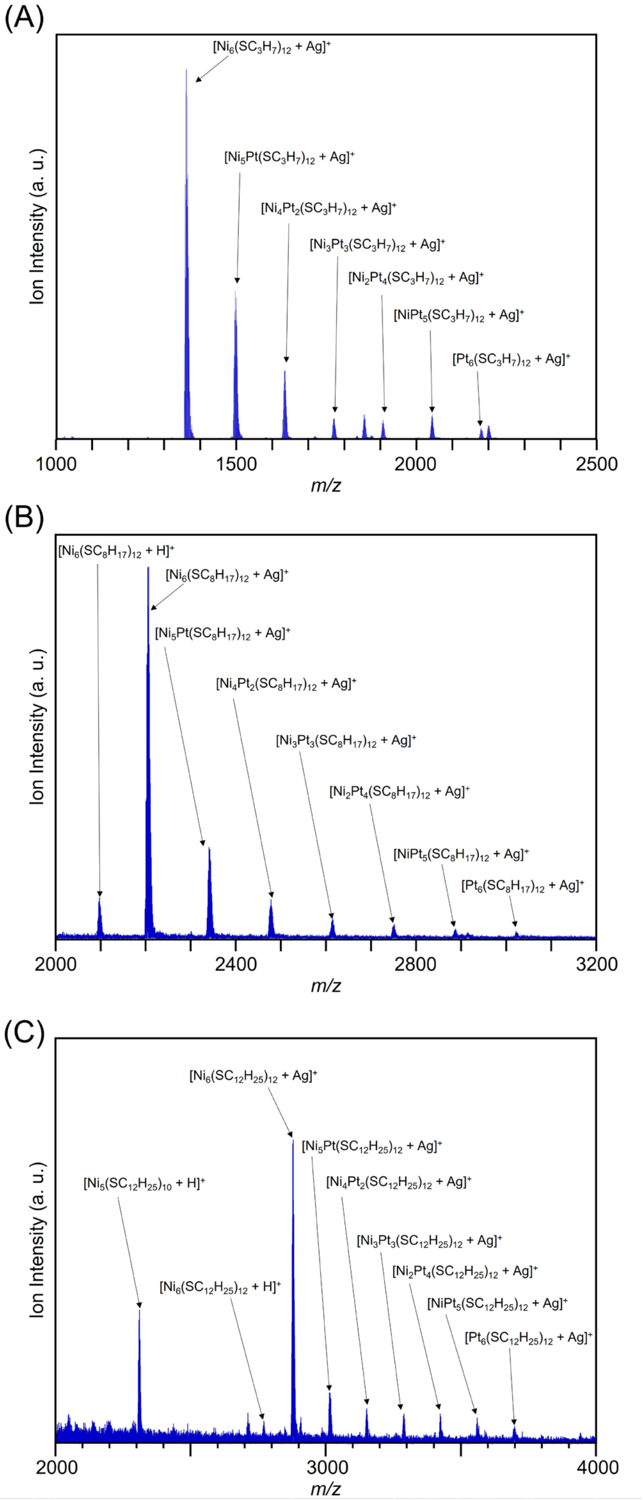

Figure S1. Positive-ion MALDI mass spectra of synthesized (A) **2**, (B) **3** and (C) **4**.

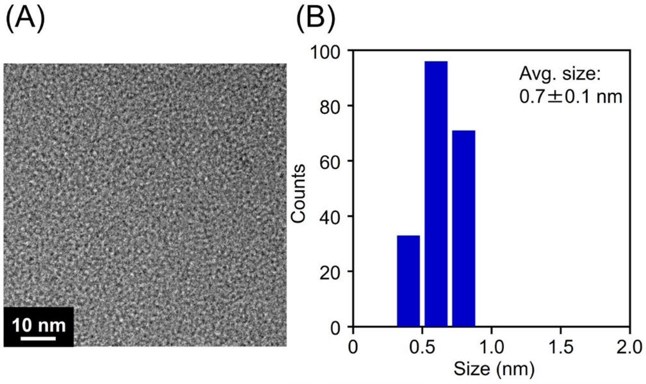

Figure S2. (A) The TEM image and (B) resulting histograms of the particle-size distribution of synthesized **1**.

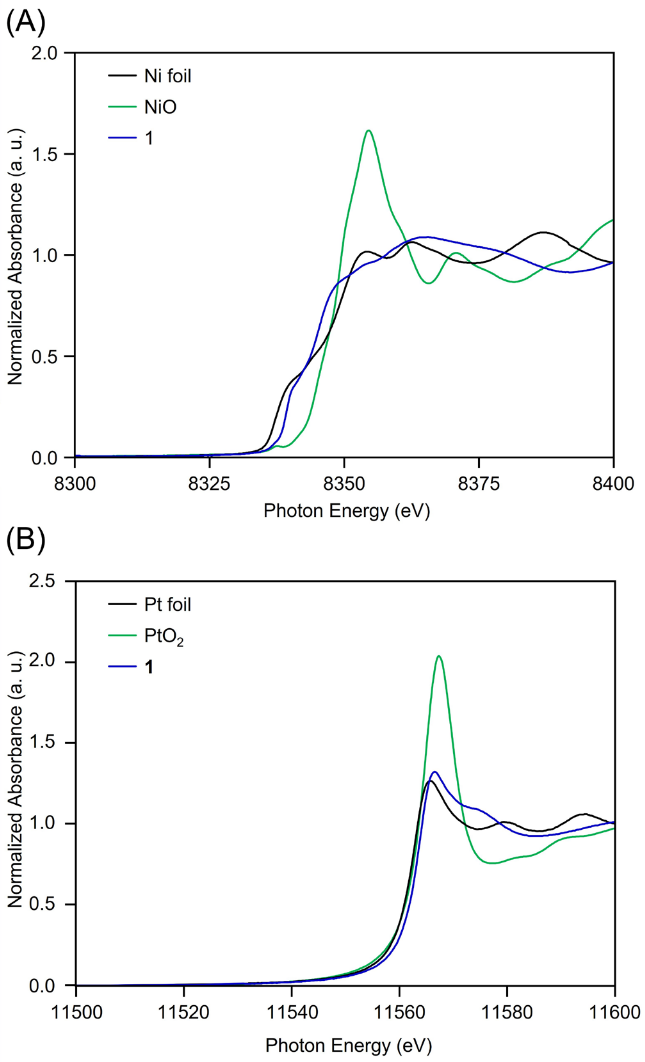

Figure S3. Results of (A) Ni K-edge and (B) Pt L_3-edge XANES spectra for synthesized **1** together with Ni foil and NiO powder in (A) and Pt foil and PtO_2 powder in (B) as a standard sample.

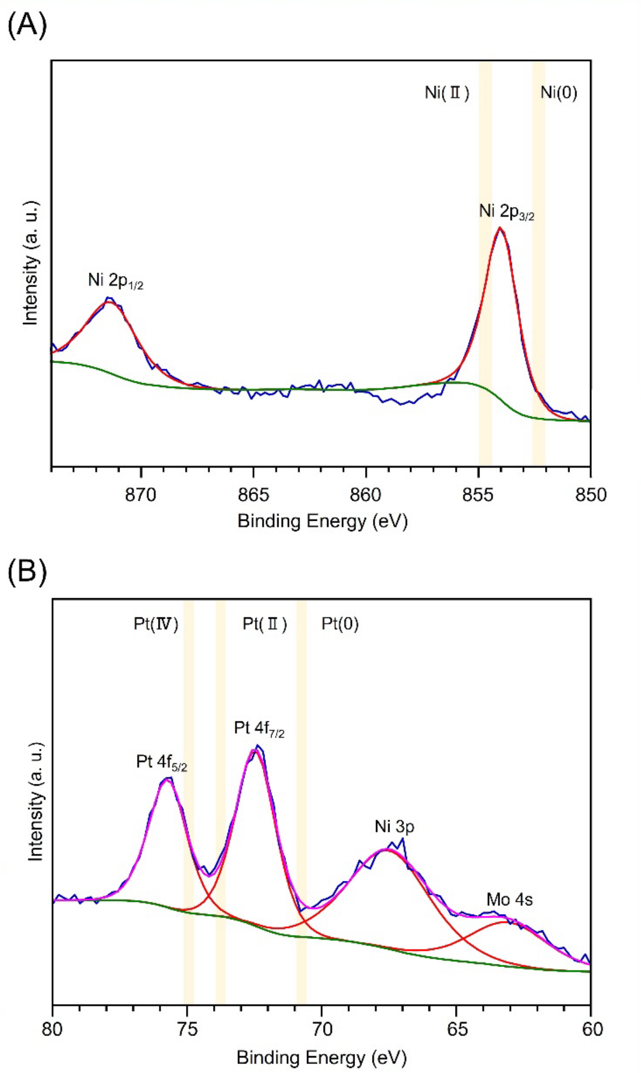

Figure S4. Results of (A) Ni $2p_{3/2}$ and (B) Pt $4f_{7/2}$ XPS spectra for $Ni_xPt_{6-x}(PET)_{12}$ (x = 0–6).

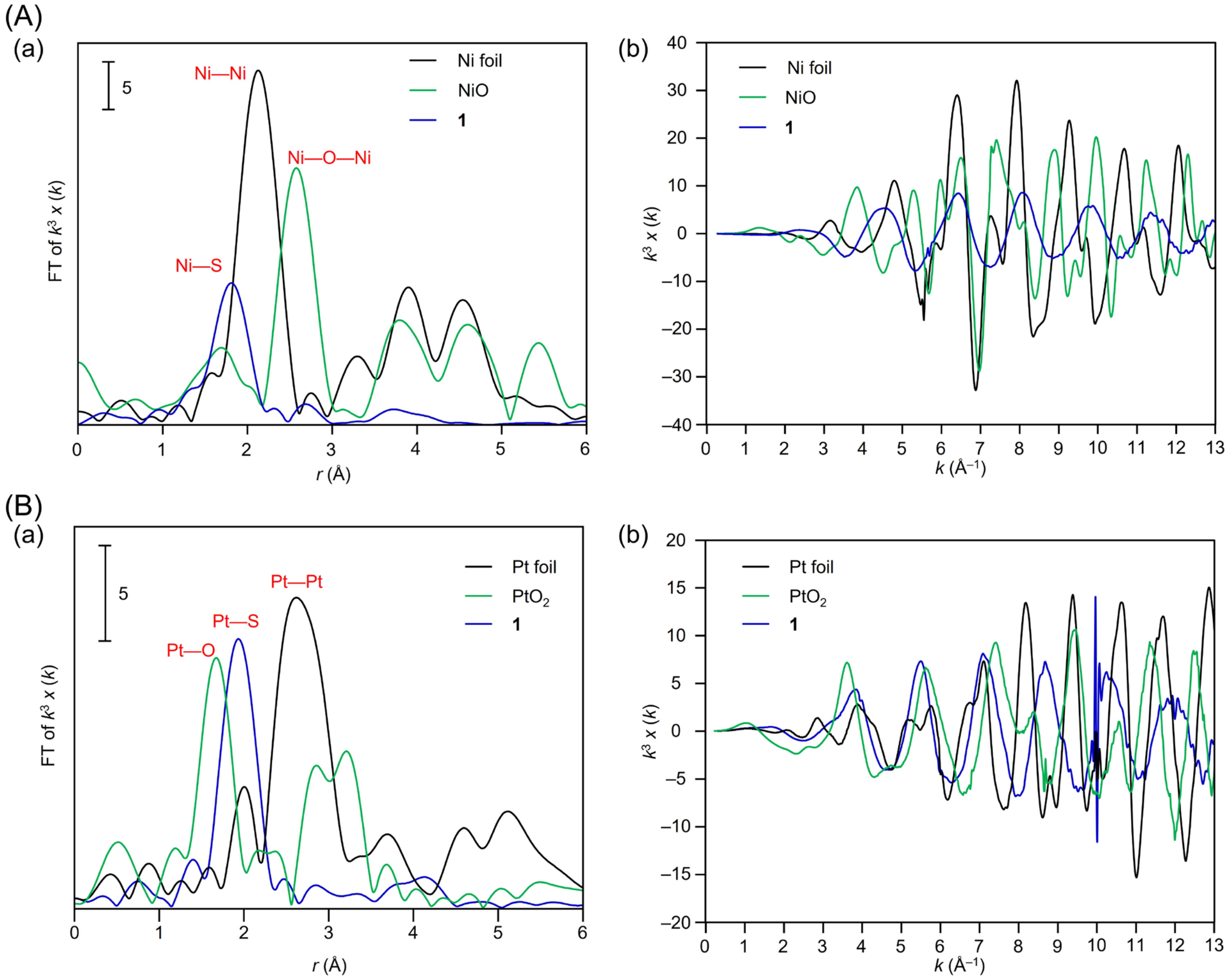

Figure S5. Results of (A) Ni K-edge and (B) Pt L_3-edge (a) FT-EXAFS and (b) EXAFS spectra for synthesized **1** together with Ni foil and NiO powder in (A) and Pt foil and PtO_2 powder in (B) as a standard sample. In (Aa) and (Ba), the peaks at 1.8 and 1.9 Å are assigned to Ni−S and Pt−S bonds, respectively. [11, 12]

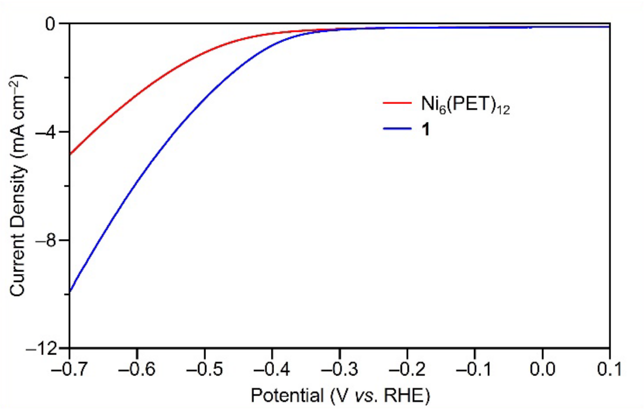

Figure S6. Electrocatalytic hydrogen evolution activity of **1** and pure $Ni_6(PET)_{12}$.

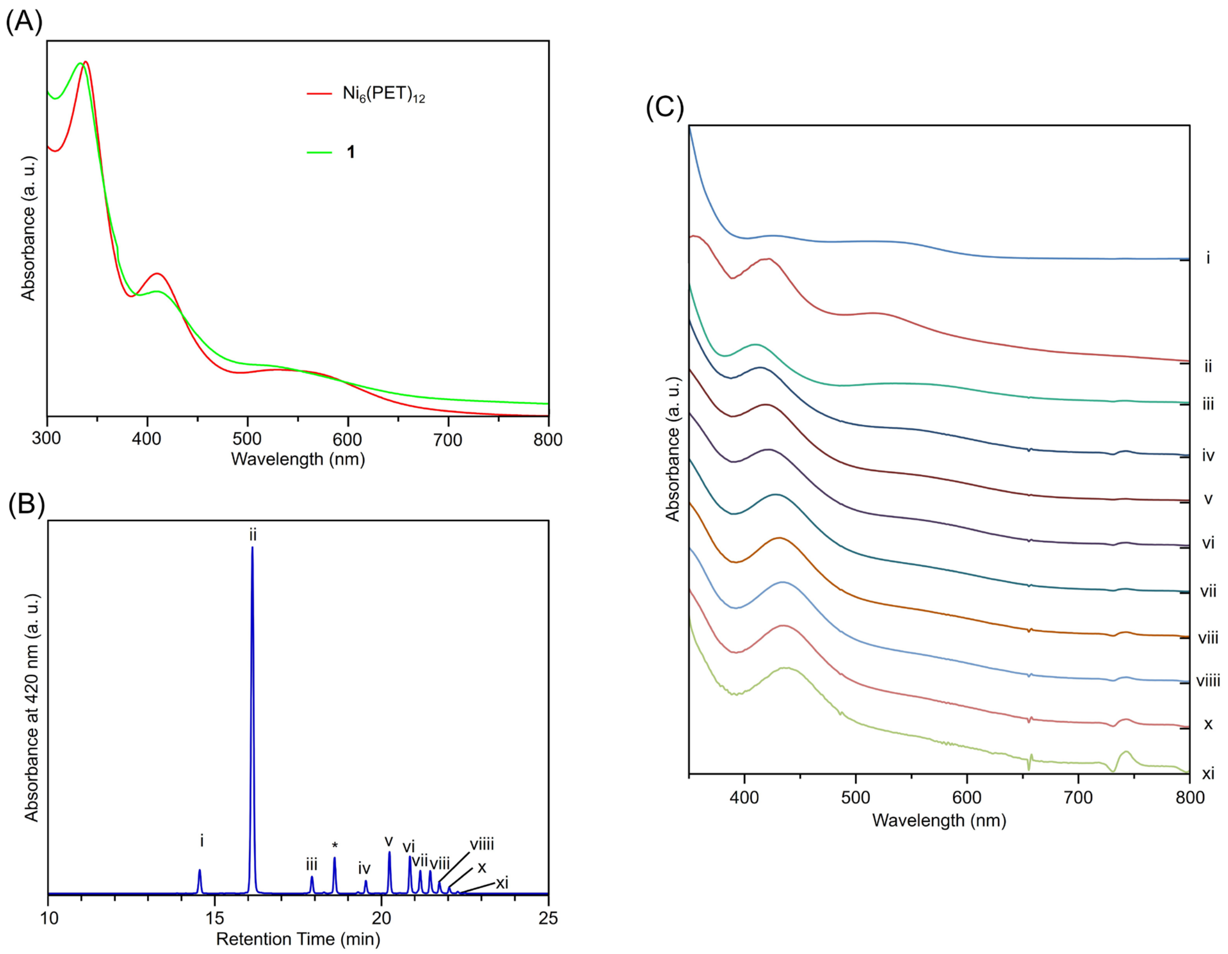

Figure S7. (A) Optical absorbance spectra of **1** and pure $Ni_6(PET)_{12}$, and (B) RP-HPLC chromatogram of **1** and (C) UV-vis optical absorption spectra of the peak (**i**–**xi**) obtained by PDA detector attached to RP-HPLC apparatus. In (B), * indicates unknown compounds.

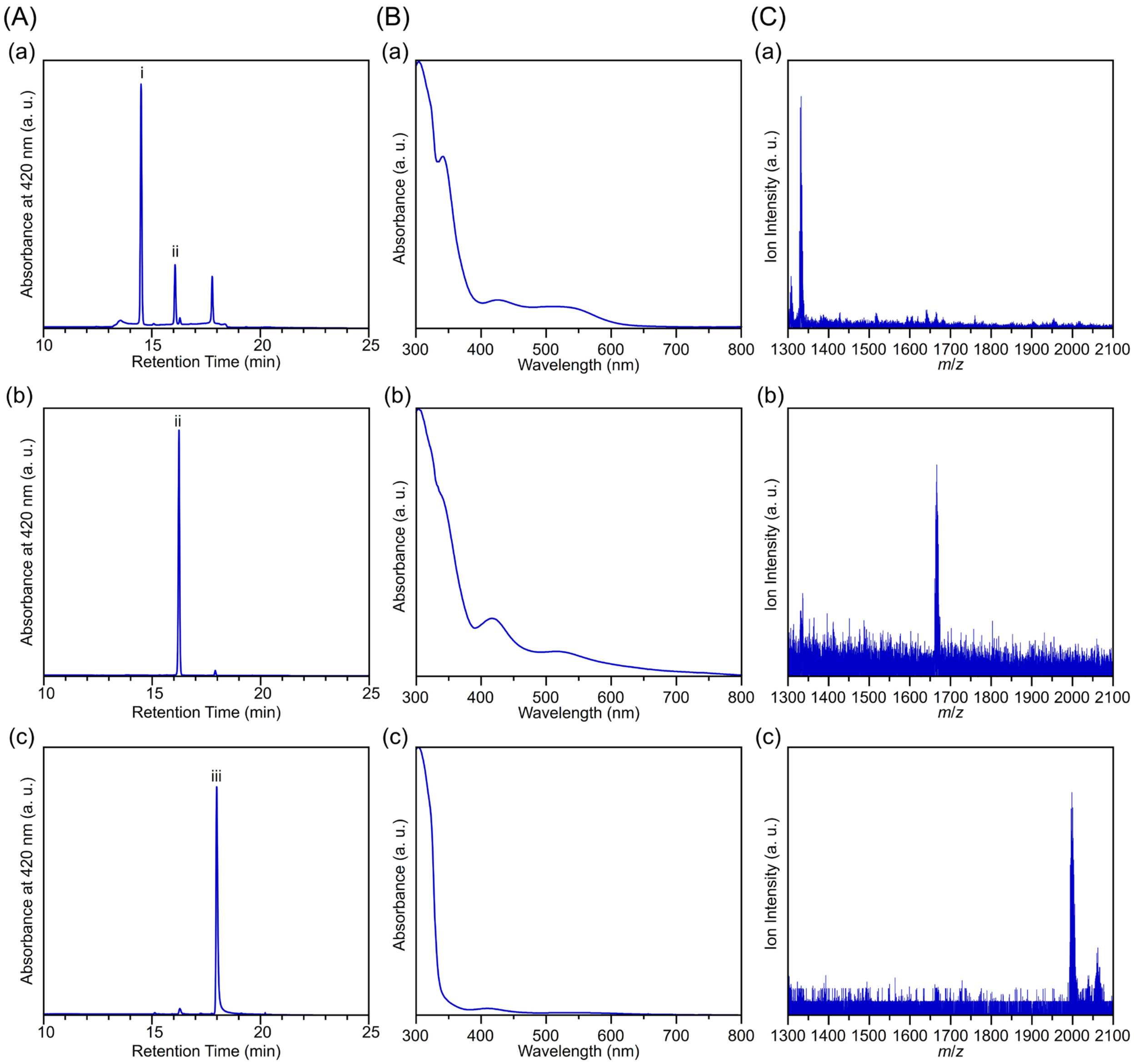

Figure S8. (A) RP-HPLC chromatograms of (a) $Ni_4(PET)_8$, (b) $Ni_5(PET)_{10}$ and (c) $Ni_6(PET)_{12}$, and (B) optical absorbance spectra of (a) peak **i** for $Ni_4(PET)_8$, (b) peak **ii** for $Ni_5(PET)_{10}$ and (c) peak **iii** for $Ni_6(PET)_{12}$ obtained with a PDA detector attached to an RP-HPLC apparatus. (C) Positive-ion MALDI mass spectra of (a) peak **i** for $Ni_4(PET)_8$, (b) peak **ii** for $Ni_5(PET)_{10}$ and (c) peak **iii** for $Ni_6(PET)_{12}$. The peaks obtained at different retention times for each NC originate from impurities introduced during TLC separation.

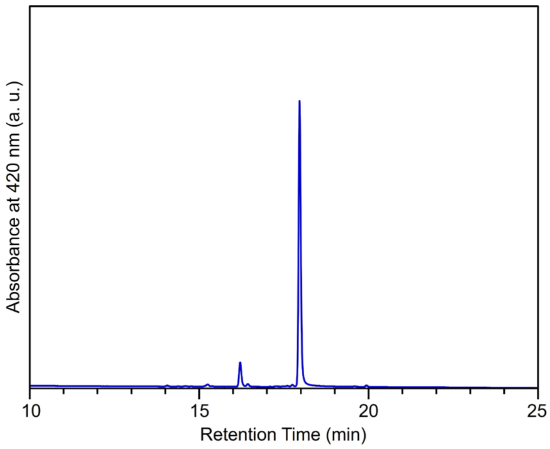

Figure S9. RP-HPLC chromatogram of synthesized **5** by metal exchange from $Ni_6(PET)_{12}$. In the metal exchange of adding Pt ions to $Ni_6(PET)_{12}$, almost no alloy TNC was obtained because of the high stability of $Ni_6(PET)_{12}$, and it is assumed that unstable species generated during the reaction produced $Ni_5(PET)_{10}$ (peak **ii**).

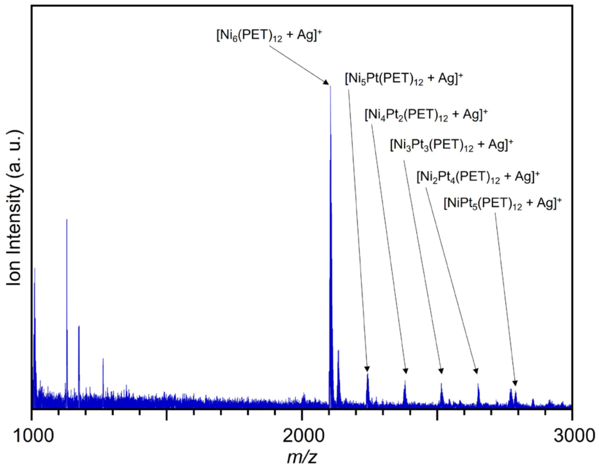

Figure S10. Positive-ion MALDI mass spectra of separated products for peak **v** in Figure S7.

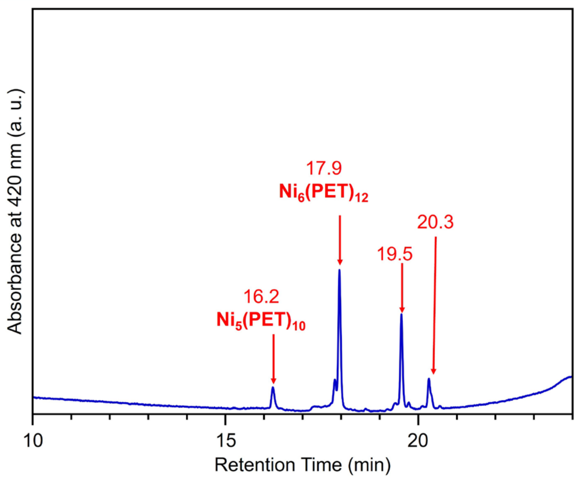

Figure S11. RP-HPLC chromatogram of separated products for peak **v** from **1** in Figure S7.

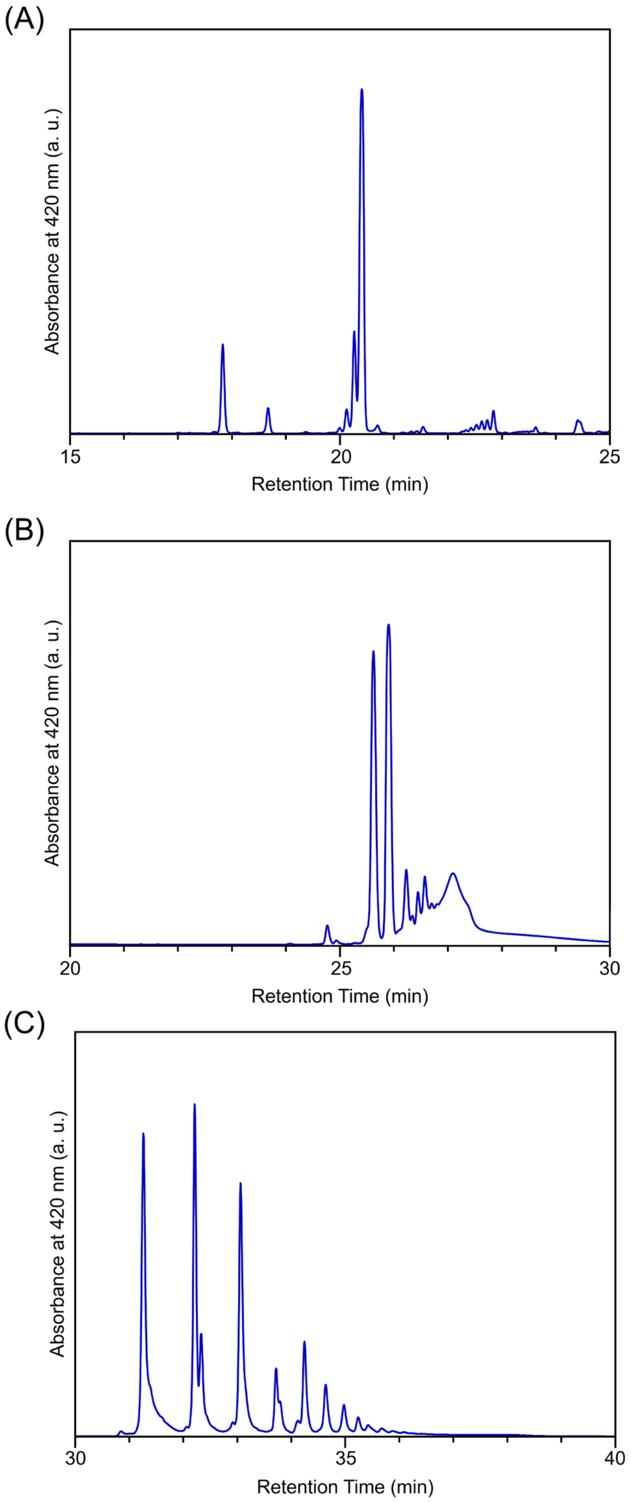

Figure S12. RP-HPLC chromatogram of synthesized (A) **2** [$Ni_xPt_{6-x}(SC_3H_7)_{12}$] and (B) **3** [$Ni_xPt_{6-x}(SC_8H_{17})_{12}$] (C) **4** [$Ni_xPt_{6-x}(SC_{12}H_{25})_{12}$].

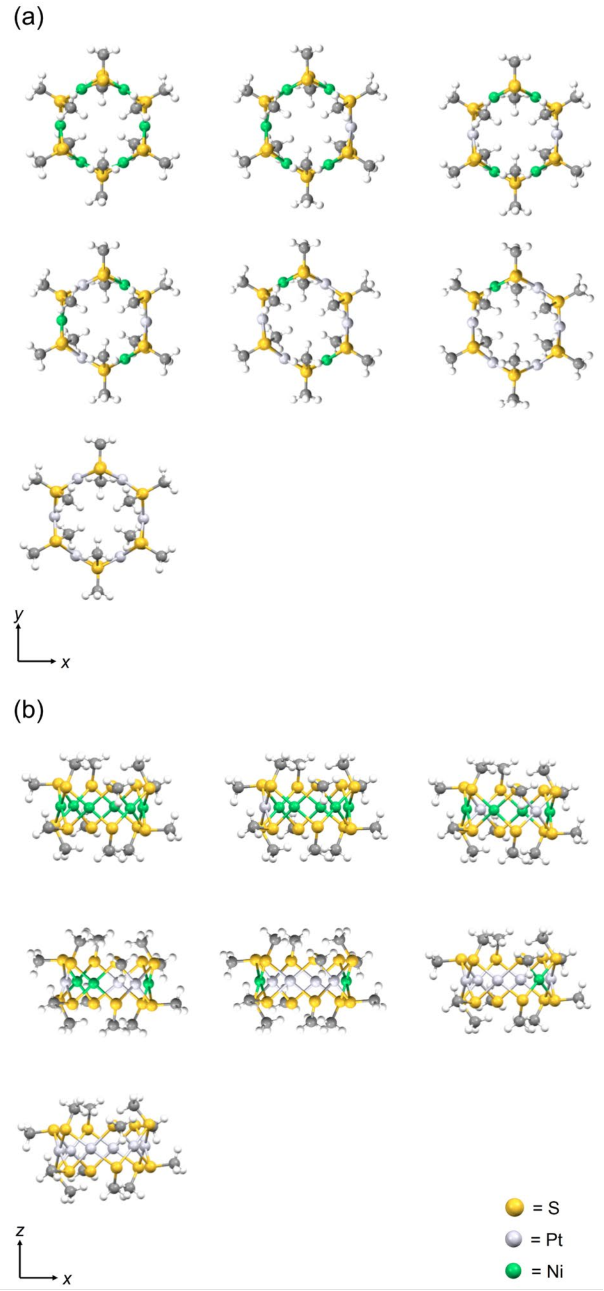

Figure S13. Geometric structures for $[Ni_xPt_{6-x}(MT)_{12}]^0$ determined by DFT calculations from (a) top and (b) side view. Ni: light green, Pt: white, S: yellow, carbon: dark gray, and hydrogen: light gray.

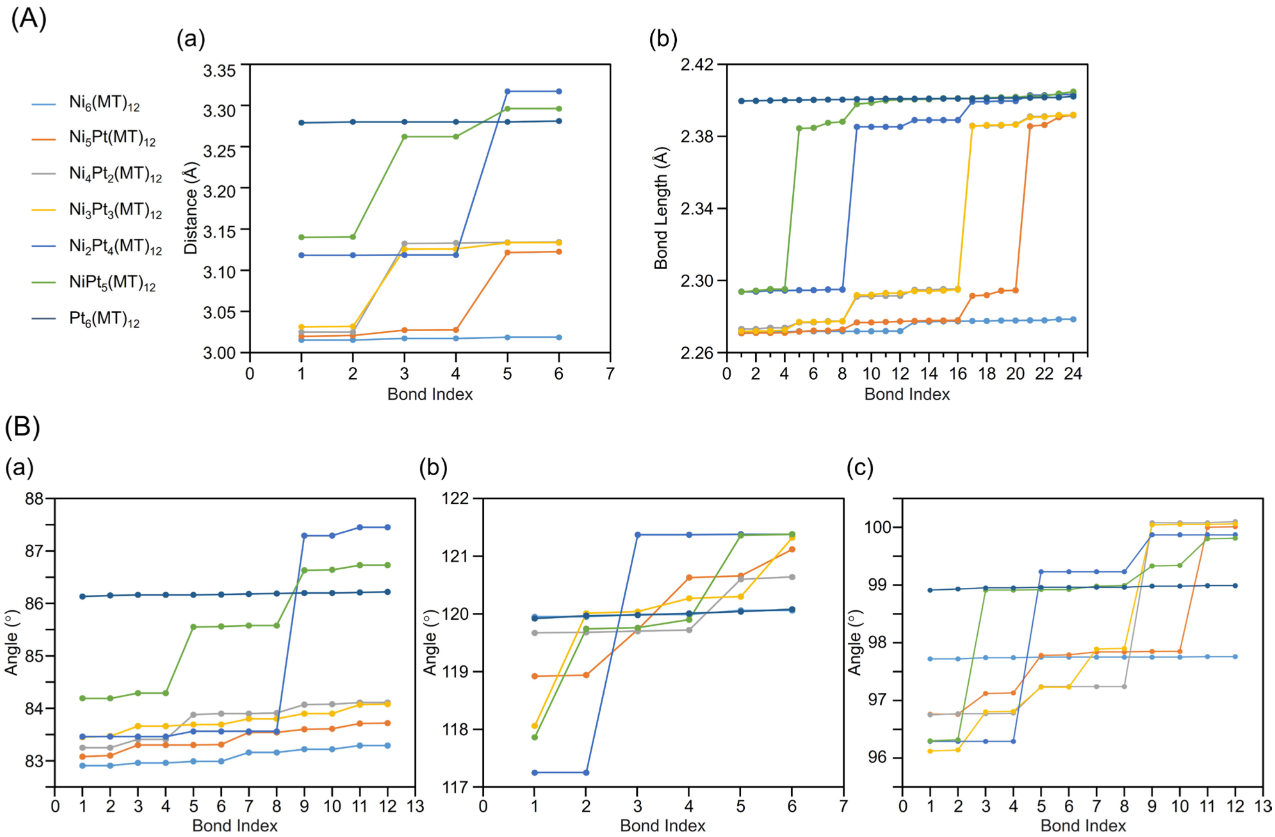

Figure S14. (A) Bond length [(a) M–M distances and (b) M–S bonds] and (B) bond angles [(a) M–S–M, (b) M–M–M and (c) S–M–S angles] from an analysis of geometric structures for $[Ni_xPt_{6-x}(MT)_{12}]^0$ determined by DFT calculations from Figure S10.

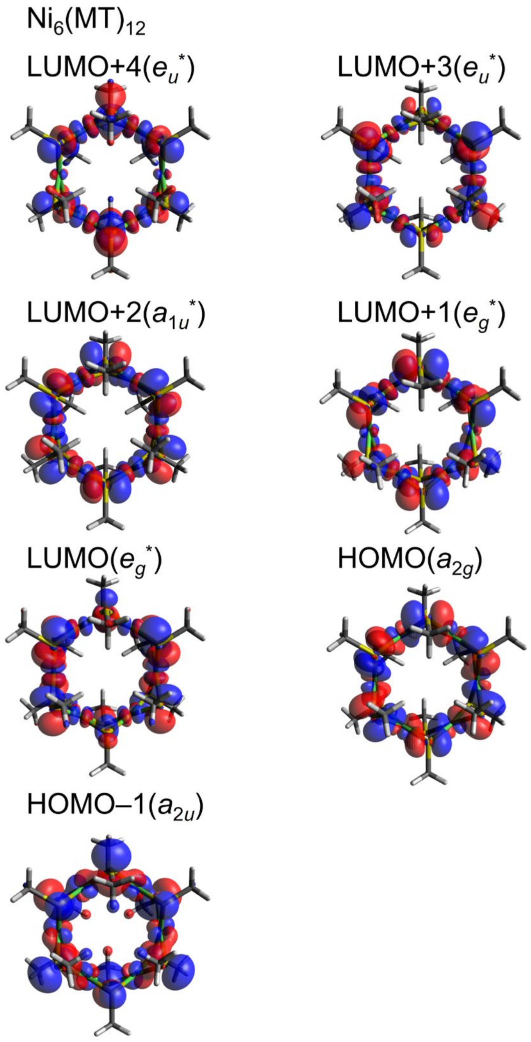

Figure S15. Molecular orbitals of $[Ni_6(MT)_{12}]^0$ determined by DFT calculations. Ni: light green, S: yellow, carbon: dark gray, and hydrogen: light gray.

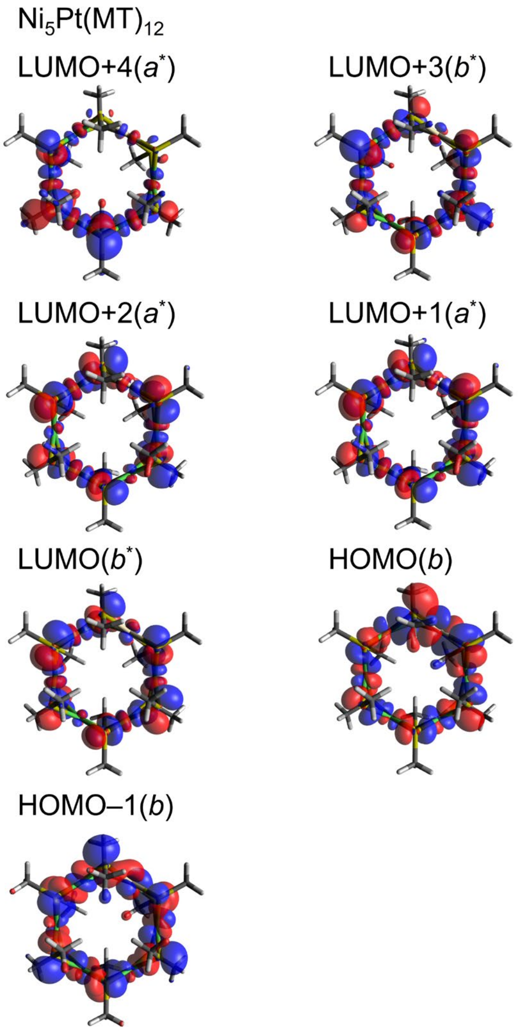

Figure S16. Molecular orbitals of $[Ni_5Pt(MT)_{12}]^0$ determined by DFT calculations. Ni: light green, Pt: white, S: yellow, carbon: dark gray, and hydrogen: light gray.

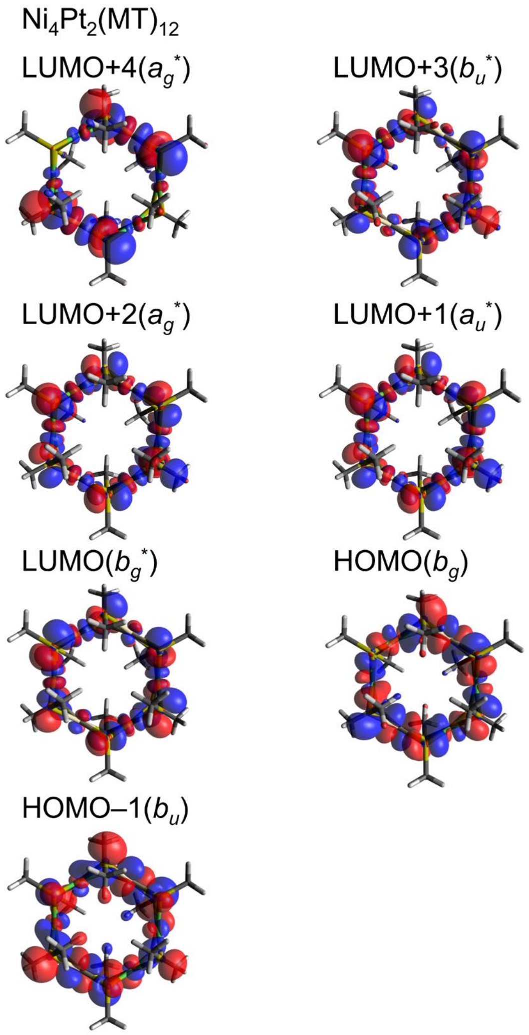

Figure S17. Molecular orbitals of $[Ni_4Pt_2(MT)_{12}]^0$ determined by DFT calculations. Ni: light green, Pt: white, S: yellow, carbon: dark gray, and hydrogen: light gray.

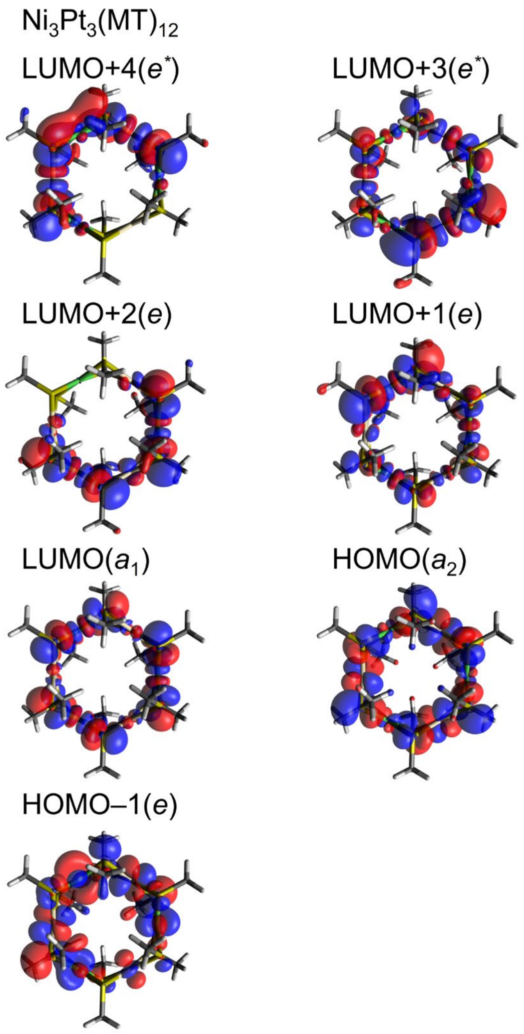

Figure S18. Molecular orbitals of $[Ni_3Pt_3(MT)_{12}]^0$ determined by DFT calculations. Ni: light green, Pt: white, S: yellow, carbon: dark gray, and hydrogen: light gray.

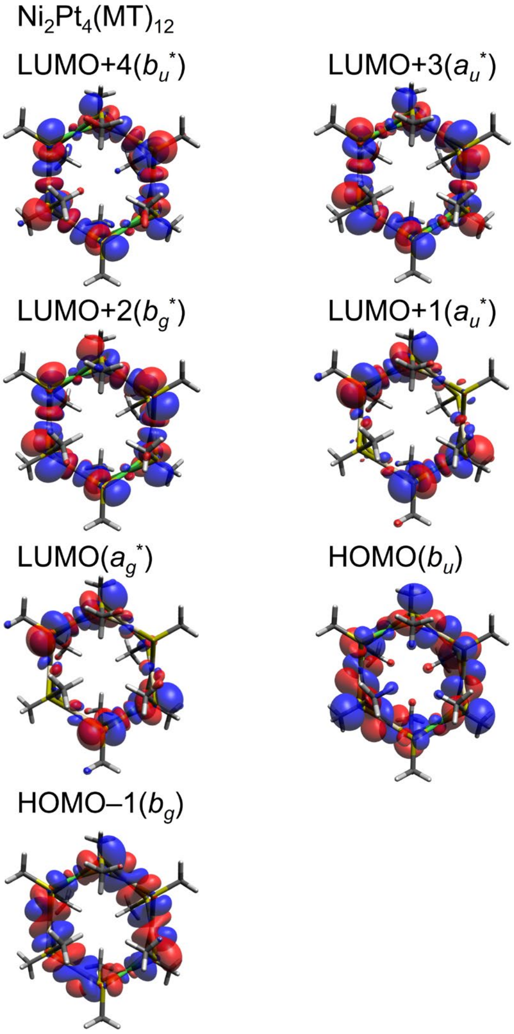

Figure S19. Molecular orbitals of $[Ni_2Pt_4(MT)_{12}]^0$ determined by DFT calculations. Ni: light green, Pt: white, S: yellow, carbon: dark gray, and hydrogen: light gray.

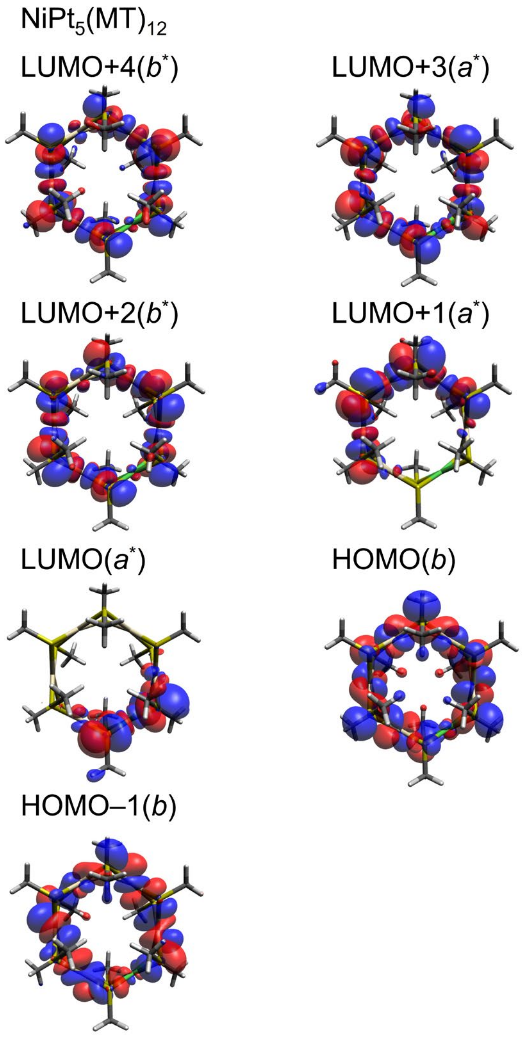

Figure S20. Molecular orbitals of $[NiPt_5(MT)_{12}]^0$ determined by DFT calculations. Ni: light green, Pt: white, S: yellow, carbon: dark gray, and hydrogen: light gray.

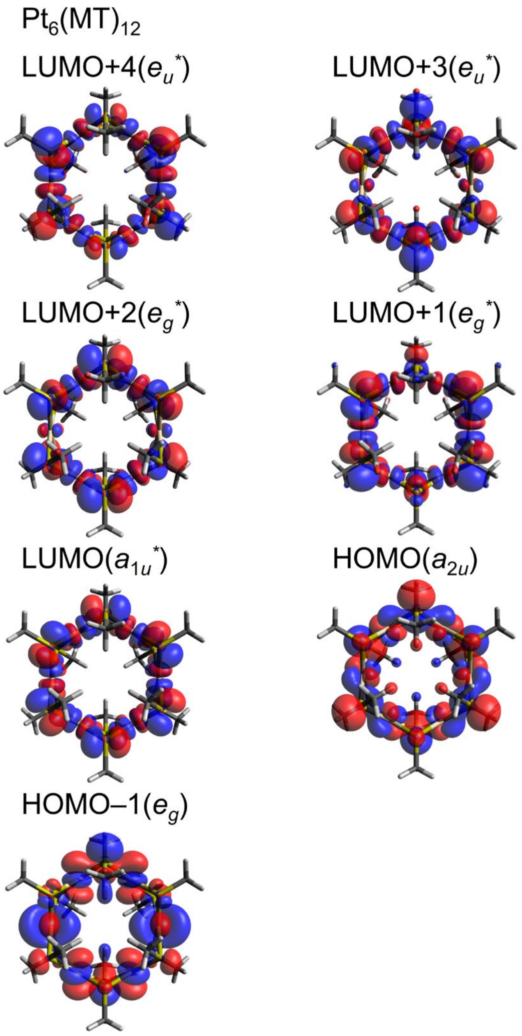

Figure S21. Molecular orbitals of $[Pt_6(MT)_{12}]^0$ determined by DFT calculations. Pt: white, S: yellow, carbon: dark gray, and hydrogen: light gray.

6. References

[1] Asakura, H.; Yamazoe, S.; Misumi, T.; Fujita, A.; Tsukuda, T.; Tanaka, T. xTunes: A New XAS Processing Tool for Detailed and On-The-Fly Analysis. *Radiat. Phys. Chem.* **2020**, *175*, 108270.

[2] Frisch, M. J.; Trucks, G. W.; Schlegel, H. B.; Scuseria, G. E.; Robb, M. A.; Cheeseman, J. R.; Scalmani, G.; Barone, V.; Petersson, G. A.; Nakatsuji, H.; Li, X.; Caricato, M.; Marenich, A. V.; Bloino, J.; Janesko, B. G.; Gomperts, R.; Mennucci, B.; Hratchian, H. P.; Ortiz, J. V.; Izmaylov, A. F.; Sonnenberg, J. L.; Williams-Young, D.; Ding, F.; Lipparini, F.; Egidi, F.; Goings, J.; Peng, B.; Petrone, A.; Henderson, T.; Ranasinghe, D.; Zakrzewski, V. G.; Gao, J.; Rega, N.; Zheng, G.; Liang, W.; Hada, M.; Ehara, M.; Toyota, K.; Fukuda, R.; Hasegawa, J.; Ishida, M.; Nakajima, T.; Honda, Y.; Kitao, O.; Nakai, H.; Vreven, T.; Throssell, K.; Montgomery, J. A., Jr.; Peralta, J. E.; Ogliaro, F.; Bearpark, M. J.; Heyd, J. J.; Brothers, E. N.; Kudin, K. N.; Staroverov, V. N.; Keith, T. A.; Kobayashi, R.; Normand, J.; Raghavachari, K.; Rendell, A. P.; Burant, J. C.; Iyengar, S. S.; Tomasi, J.; Cossi, M.; Millam, J. M.; Klene, M.; Adamo, C.; Cammi, R.; Ochterski, J. W.; Martin, R. L.; Morokuma, K.; Farkas, O.; Foresman, J. B.; Fox, D. J. Gaussian 16, Revision B.01. Gaussian, Inc., Wallingford CT, 2016.

[3] Becke, A. D. Density-Functional Exchange-Energy Approximation with Correct Asymptotic Behavior. *Phys. Rev. A* **1988**, *38*, 3098−3100.

[4] Weigend, F.; Ahlrichs, R. Balanced Basis Sets of Split Valence, Triple Zeta Valence and Quadruple Zeta Valence Quality for H to Rn: Design and Assessment of Accuracy. *Phys. Chem. Chem. Phys.* **2005**, *7*, 3297−3305.

[5] Francl, M. M.; Pietro, W. J.; Hehre, W. J.; Stephen Binkley, J.; Gordon, M. S.; DeFrees, D. J.; Pople, J. A. Self-Consistent Molecular Orbital Methods. XXIII. A Polarization-Type Basis Set for Second-Row Elements. *J. Chem. Phys.* **1982**, *77*, 3654−3665.

[6] Andrae, D.; Häußermann, U.; Dolg, M.; Stoll, H.; Preuß, H. Energy-Adjusted *ab Initio* Pseudopotentials for the Second and Third Row Transition Elements. *Theor. Chim. Acta* **1990**, *77*, 123−141.

[7] Becke, A. D. A New Mixing of Hartree–Fock and Local Density-Functional Theories. *J. Chem. Phys.* **1993**, *98*, 1372−1377.

[8] Lee, C.; Yang, W.; Parr, R. G. Development of the Colle-Salvetti Correlation-Energy Formula into a Functional of the Electron Density. *Phys. Rev. B* **1988**, *37*, 785−789.

[9] Runge, E.; Gross, E. K. U. Density-Functional Theory for Time-Dependent Systems. *Phys. Rev. Lett.* **1984**, *52*, 997−1000.

[10] Pan, Y.; Chen, J.; Gong, S.; Wang, Z. Co-Synthesis of Atomically Precise Nickel Nanoclusters and the Pseudo-Optical Gap of $Ni_4(SR)_8$. *Dalton Trans.* **2018**, *47*, 11097−11103.

[11] Li, Q.; Wang, D.; Han, C.; Ma, X.; Lu, Q.; Xing Z.; Yang, X. Construction of Amorphous Interface in an Interwoven NiS/NiS_2 Structure for Enhanced Overall Water Splitting. *J. Mater. Chem. A* **2018**, *6*, 8233–8237.

[12] Imaoka, T.; Akanuma, Y.; Haruta, N.; Tsuchiya, S.; Ishihara, K.; Okayasu, T.; Chun, W.-J.; Takahashi, M.; Yamamoto, K. Platinum Clusters with Precise Numbers of Atoms for Preparative-Scale Catalysis. *Nat. Commun.* **2017**, *8*, 688.
